# Elucidating the Kinetics and Thermodynamics of Organic Vapor Phase Infiltration and Molecular Layer Deposition for Dissolution Resilient Polymers

**DOI:** 10.1021/acs.chemmater.5c01522

**Published:** 2025-10-03

**Authors:** Brian C. Welch, Bratin Sengupta, Ruoke Cai, Vepa Rozyyev, Eitan I. Feldman, Anil Mane, Alon Grinberg Dana, Jeffrey W. Elam, Tamar Segal-Peretz

**Affiliations:** † Department of Chemical Engineering Technion − 26747 Israel Institute of Technology, Haifa 3200003, Israel; ‡ Applied Materials Division, 1291 Argonne National Laboratory, Lemont, Illinois 60439, United States; § Northwestern Center for Water Research, Northwestern University, Evanston, Illinois 60201, United States; ∥ The Interdisciplinary Program in Polymer Engineering, Technion − Israel Institute of Technology, Haifa 3200003, Israel; ⊥ Department of Chemical and Biomolecular Engineering, 3990 Rice University, Houston, Texas 77005, United States

## Abstract

Vapor phase chemical synthesis techniques, such as atomic layer deposition (ALD) and vapor phase infiltration (VPI), enable molecular-level tailoring of polymeric materials through deposition or incorporation of inorganic components. However, benefits are often paired with compromised mechanical stability and organic–inorganic bonds that are prone to degradation via hydrolysis. To address these limitations, we investigate all-organic VPI and molecular layer deposition (MLD) chemistries as a strategy for enhancing polymer properties. We examine the reaction-diffusion kinetics and thermodynamic behavior of three aromatic step-growth polymerization reactions: polyamide (isophthaloyl chloride + m-phenylenediamine, MPD), polyurea (1,4-phenylene diisocyanate + MPD), and polyimine (terephthalaldehyde + MPD). Their material growth occurs via MLD at the surfaces of nonabsorbing silicon and zirconia. Organic VPI occurs within the bulk of nucleophile-rich polyvinyl alcohol (PVA), but not through physical entrapment in unreactive polystyrene and poly­(methyl methacrylate). Using a reaction-diffusion model, we quantify diffusion-limited polyamide and reaction-limited polyurea nucleation behavior in PVA, identifying key parameters: diffusivity, reaction rate, and Damköhler number. Unlike inorganic alumina treatment, organic modification enhances dissolution-resistance in PVA, preserving polymer integrity and resisting hydrolysis even in harsh pH 13 solutions. This study demonstrates the potential of all-organic material deposition for synthesizing novel polymers with improved durability and solvent resilience.

## Graphical abstract

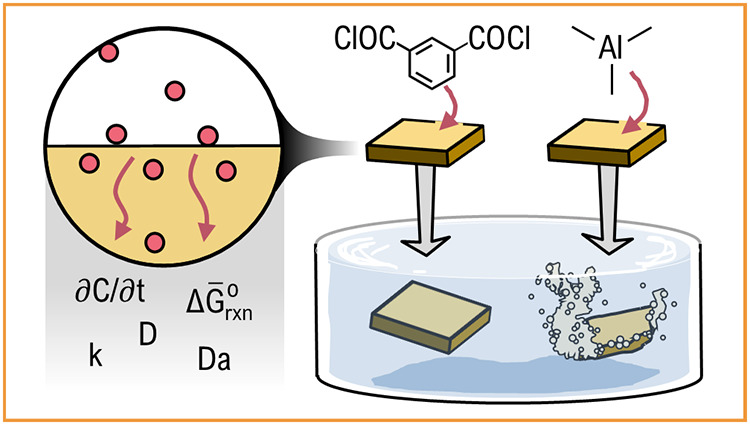

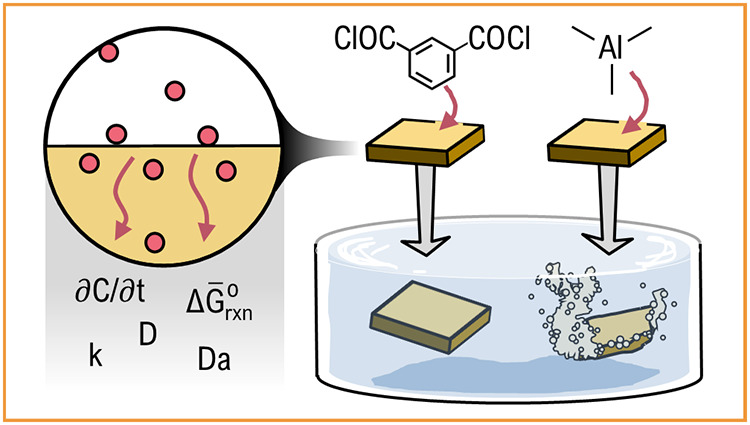

## 1 Introduction

Organometallic reactant vapors are effective for transforming synthetic polymers and biological materials and can be utilized to increase toughness and provide solvent resistance. In a process known as vapor phase infiltration (VPI), the reactants penetrate the bulk of the starting polymer material in controlled steps, transforming it through the deposition of inorganic clusters within the free volume of the polymer and cross-links between polymer chains.^1^

However, VPI (also known as sequential infiltration synthesis, SIS) faces drawbacks. The gains of modification may be temporary, as hybrid materials grown with metalorganic precursors are known to be susceptible to hydrolysis when exposed to water – even humidity.^2,3^ And while increased toughness has been demonstrated in seminal works,^4,5^ materials treated with metalorganic vapors often exhibit increased stiffness together with increased brittleness – an outcome that challenges efforts to tune bulk material properties through VPI or apply functional surface coatings with atomic layer deposition (ALD) or chemical vapor deposition.^6^ We hypothesize that all-organic reactants can circumvent these shortcomings to enable enhancement of polymers with molecule-scale control.

While the category of infiltrating organic vapor reactants remains largely unexplored, such reactants have been studied extensively for thin film deposition via all-organic molecular layer deposition (MLD). This technique of stepwise polymerization enables molecule-scale control over thickness and composition, and has been leveraged for creating time-release coatings for pharmaceutical powders,^7,8^ selective layers of polymeric membranes,^9–12^ and electronically conductive, yet flexible surfaces of sponge electrodes.^13^

The MLD process is similar to that of atomic layer deposition (ALD), but instead of inorganic films, MLD can deposit step-growth polymer films by repeating two isolated steps (Figure 1a).^14^ In the first step, an electrophilic monomer (E) is vaporized and introduced to the sample surface under a controlled, inert, medium-vacuum environment. A substitution reaction occurs, covalently binding the monomers to nucleophilic surface groups (e.g., hydroxyls). The monomer precursors typically contain multiple functional groups of the same type of electrophile, limiting deposition to a single layer that refunctionalizes the surface. This step is concluded by purging excess precursor and reaction byproducts. In the second step, a nucleophilic monomer (N) is introduced, reacting with the electrophilic surface functional groups and, again, forming a single layer of material in a self-limited manner. After purging this second step the MLD cycle is complete, and the first monomer may be used again to continue film growth. By repeating these two steps, a polymer film is grown with Angstrom-scale precision (Figure 1b).

**Figure 1:**
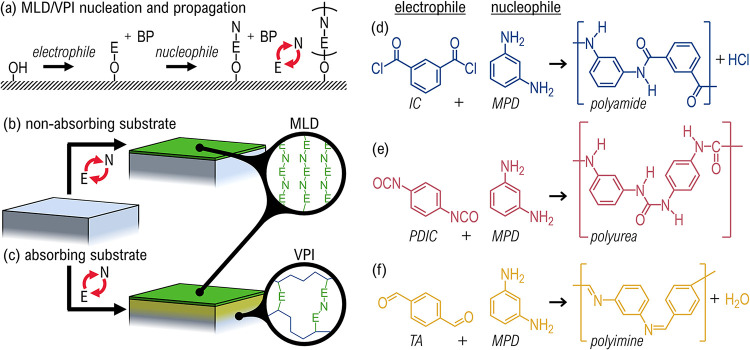
All organic MLD and VPI on and within polymers. (a) Self-limited polymer MLD growth occurs through two isolated reaction steps involving electrophile (E), and nucleophile (N) vapor precursors. Excess reactant and byproduct (BP) vapors are purged between each step. (b) MLD occurs at the surface of a sample that cannot absorb the precursor. (c) Substrates that absorb the precursors have surface film growth via MLD, and subsurface material deposition/cross-link formation via VPI. (d–f) MLD reactions in this study representing three common MLD-grown polymers: polyamide, polyurea, and polyimine. (d) Amide polycondensation between isophthaloyl chloride (IC) and m-phenylenediamine (MPD) with HCl as a byproduct. (e) Urea polyaddition between 1,4-phenylene diisocyanate (PDIC) and MPD with no byproduct. (f) Imine polycondensation between terephthalaldehyde (TA) and MPD with H_2_O as a byproduct.

Possible electrophiles for MLD include (i) acyl chlorides, (ii) isocyanates, (iii) anhydrides, and (iv) aldehydes, while nucleophiles are typically alcohols or amines. Among these eight pairings, polyamides (acyl chloride + amine), polyureas (isocyanate + amine), and polyimides (anhydride + amine) are the most widely studied, likely due to their favorable reactivity.^15^

Polymer substrates are absorbent to organic vapor phase reactants, and present unique challenges when monomers diffuse into the bulk of the material, complicating the surface-controlled nature of MLD (Figure 1c). A related phenomenon has been studied with ALD, where inorganic precursors undergo Lewis acid–base interactions within the bulk of the sample, causing subsurface deposition.^16^ Initially perceived as a challenge for polymer surface modification, this behavior was reimagined as a useful feature, enabling the modification of polymer properties throughout the thickness of absorbing substrates by the incorporation of inorganic material.^4,17^ This approach, termed VPI, copies the stepwise ALD procedure, but uses prolonged steps to encourage subsurface interactions.

VPI has since been used to enhance resilience against solvents. For example, VPI modification using trimethylaluminum (TMA) and H_2_O precursors has conferred solvent resistance in polycarbonate, PIM-1, Poly­(methyl methacrylate) (PMMA), and poly­(styrene-random-methyl methacrylate) (P­(S-*r*-MMA)) against various organic solvents known to dissolve the unmodified polymers.^18–21^

In this study, we investigate vapor phase chemistry within polymer substrates by characterizing and comparing the reaction and transport kinetics between organic and inorganic VPI. We examine three representative chemistries- polyamide, polyurea, and polyimine (Figure 1 d–f). These reactions use aromatic electrophiles, including acyl chlorides, isocyanates, and aldehydes, together with an aromatic amine nucleophile. Notably, the polyamide of Figure 1d corresponds to the same chemistry used to produce the flame-resistant aramid, Nomex, which has not been previously demonstrated with MLD, to our knowledge. Anhydrides are a fourth representative electrophile often used in MLD to form polyimides, but they are excluded from this study due to the high processing temperatures required.

We begin by characterizing MLD on nonabsorbing substrates using *in situ* ellipsometry and Fourier transform infrared spectroscopy (FTIR) to analyze growth without the effects of subsurface VPI. We then perform these chemistries on polymeric substrates, including polyvinyl alcohol (PVA), PMMA, and polystyrene (PS), using *in situ* ellipsometry to assess VPI growth. To further analyze VPI in PVA, we employ X-ray photoelectron spectroscopy (XPS) and quartz crystal microbalance (QCM) measurements to quantify growth and penetration depth.

PVA is an excellent substrate for studying organic VPI. This water soluble, hydrophilic vinyl polymer is abundant in hydroxyl groups that act as nucleophiles. Cross-linking PVA is widely studied as a means to control water stability for applications including selective membranes, tissue engineering, drug delivery, and textile engineering.^22,23^

We implement a quantitative framework for describing the reaction-diffusion behavior of each electrophile in PVA. The analysis is based on the Ren-McGuinness reaction-diffusion transport model and characterizes a VPI system according to three nondimensional parameters: (1) relative rates of reaction and diffusion (Damköhler number, *Da*), (2) the hindrance to diffusion caused by reactions (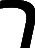, Hebrew letter “resh”), and (3) the relative concentrations of precursor and functional groups (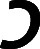, Hebrew letter “kaf”).^6,24^

Finally, we harness organic MLD/VPI for enhancing the solvent resistance of polymers. We demonstrate that organic MLD/VPI creates hydrolysis-resilient bonds that impart solvent resistance to PVA. While PVA samples modified with inorganic (TMA-H_2_O) treatment are susceptible to dissolution in water, samples modified with organic precursors remain stable, even in highly alkaline (pH 13) KOH solutions. These results demonstrate that the shortcomings of VPI can be addressed by replacing inorganic chemistries with chemically stable, mechanically robust organic polymer chemistries.

## 2 Materials and Methods

### 2.1 Polymers and Precursors

Isophthaloyl chloride (IC) (>99%), terephthalaldehyde (TA) (>99%), m-Phenylenediamine (MPD) (>99%), 1,4-phenylene diisocyanate (PDIC), HPLC-grade water, Mowiol 4–88, Mowiol 8–88, PS (MW ∼192 kg/mol), and PMMA were obtained from Sigma-Aldrich. TMA (>98%) was purchased from STREM. For QCM measurements, PDIC (>98%) was obtained from Angene and MPD (>99%) was obtained from Acros Organics. The zirconium oxide (ZrO_2_) nanoparticles (∼20 nm diameter) used in FTIR measurements were purchased from US Research Nanomaterials Inc. The steel meshes used for FTIR measurement were obtained from Fotofab, Inc. and have a thickness of 50 μm with 50 μm bars and 200 μm square openings. All vapor-phase reactions were conducted under inert conditions using ultrahigh purity nitrogen (99.999%). QCM crystals (750-1058-G10) were obtained from Inficon. Si(100) substrates were used with a native oxide of 15 nm. Two forms of PVA were used: Mowiol 8–88 (molecular weight ∼67 kg/mol) was used in all cases except for QCM measurements, dissolution tests, and TMA-H_2_O XPS measurements where Mowiol 4–88 (molecular weight ∼31 kg/mol) was used. Both forms contained 86.7–88.7 mol% alcohol groups, with the remainder consisting of acetate groups. The difference in molecular weight was considered unimpactful for this study.

### 2.2 Spin-Coating

Prior to spin-coating, substrates were rinsed with acetone, isopropyl alcohol, methanol, and then water. After spin-coating, samples were oven-dried at 70 °C. The conditions used for spin-coating are summarized in Table S1.

### 2.3 Vapor Phase Material Growth

Two systems were used for MLD/VPI. The first system was a custom-built, hot-wall, viscous flow reactor detailed in ref 25. This system was used to create samples for FTIR and XPS analysis and to perform *in situ* ellipsometry. Nitrogen was used as the carrier gas, with a total mass flow rate of 250 sccm and a background pressure maintained between 0.9 and 1.0 Torr. Reaction temperatures were 130 °C. Samples equilibrated in the reaction chamber under vacuum for over 30 min before the first dose. Electrophile precursors were always dosed first in each cycle, followed by MPD. Reaction conditions for all experiments performed in this system are detailed in Table S2.

The second system was a custom-built isothermal reactor. This system was used for *in situ* QCM measurements. A system temperature of 130 °C was maintained by a convection fan and electrical heaters. Similar isothermal designs are described in refs 26,27 . All precursors were stored in stainless steel containers and dosed into the reaction tube via pneumatic valves. Reactor pressure was monitored with a capacitance monometer (AA09A Baratron, MKS) while temperatures were monitored using K-type thermocouples.

### 2.4 Ellipsometry Measurements

*In situ* film thickness measurements were performed in the viscous flow reactor with a Film Sense FS-1EX ellipsometer at an incidence angle of 65°. Samples included silicon coupons and polymer films spin-coated onto silicon coupons. Data analysis was performed with Film Sense Software, Version 3.16. Initial sample thicknesses are listed in Table S3.

Ex situ film thickness measurements were performed using an α-SE ellipsometer (J.A. Woollam) and analyzed with CompleteEase software (J.A. Woollam). Parameters for the ellipsometry models are provided in Table S4.

### 2.5 Fourier Transform Infrared Spectroscopy

Transmission FTIR measurements were performed ex situ with a Nicolet 6700 FTIR spectrometer (Thermo Fisher Scientific). Each spectrum was acquired as an average of 256 scans over the spectral range of 4000–400 cm^–1^. Measurements were conducted under vacuum at room temperature, with samples given 30 min to equilibrate before measurement. Samples were prepared by pressing ZrO_2_ nanoparticles onto a steel mesh grid, then applying three MLD cycles in the viscous flow reactor. Measurements were taken both before and after MLD modification with conditions detailed in Table S2. Difference spectra were generated by subtracting the pre-MLD measurement from the post-MLD measurement. Baseline corrections for the difference spectra were applied using spline fits in OMNIC software (Thermo Fisher Scientific).

### 2.6 X-Ray Photoelectron Spectroscopy with Depth Profiling

XPS measurements were conducted using a Thermo Fisher K-Alpha+ XPS instrument, using a microfocused monochromatic Al Kα (1487 eV) X-ray source with a 400 μm spot size. XPS samples included modified polymer films that were spin-coated onto silicon coupons. Samples modified with MLD precursors were prepared in the viscous flow reactor (conditions in Table S2), while the sample modified with TMA and H_2_O was prepared in the isothermal reactor (conditions in Table S5).

For the survey scan we used a pass energy of 200 eV with a step size of 1.0 eV. The survey scan was averaged over 5 scans to obtain a high signal-to-noise ratio. High resolution scans for desired elements were conducted with a lower pass energy of 50.0 eV and smaller step size of 0.1 eV, and were averaged over 5 scans.

All XPS signals were shifted based on the reference peak for adventitious carbon at 284.6 eV. The XPS data were analyzed using Avantage software (Thermo Fisher, version 5.9931, build 0.6795). Smart background was used along with a Powell peak fitting algorithm with mixed Gaussian–Lorentzian line shapes.

### 2.7 *In situ* Quartz Crystal Microbalance Measurements

*In situ* QCM measurements were performed with an Inficon ALD QCM sensor (750-713-G4) and STM-2 monitor at a sampling rate of 10 Hz. Samples included polymer thin films spin-coated onto the quartz monitor crystals to measure mass changes during precursor exposures. Mass changes were calculated from frequency data according to the Sauerbrey equation.^28^

Prior to experiments, samples were heated in the system under vacuum overnight to achieve thermal equilibrium and remove residual moisture. For experiments with exposure times ≤1 s (“short exposure”), an inert nitrogen flow was applied to the back side of the sensor to prevent internal deposition (conditions summarized in Table S5). The flow rate was adjusted using a needle valve (BMG valve, Swagelok) until reactor pressure increased by 10% above the base pressure. No nitrogen backflow was used for experiments with precursor exposure times of 1 h (“long exposure”, conditions summarized in Table S6). For these experiments, the exposure step was performed in static mode – no N_2_ carrier gas and no active pumping during precursor exposure.

### 2.8 Thermodynamic Calculations

The thermodynamics of reactions shown in Figure 1d–f were quantified by calculating their standard Gibbs free energy of reaction (Δ*G̅*_rxn_^0^) from the Gibbs free energies of formation (Δ*G̅* ^0^_f_) of products and reactants as follows

1 $$\Delta {\mathrm{G̅}}_{\mathrm{rxn}}^{o}=\sum {\left(\Delta {\mathrm{G̅}}_{f}^{o}\right)}_{\mathrm{products}}-\sum {\left(\Delta {\mathrm{G̅}}_{f}^{o}\right)}_{\mathrm{reactants}}$$

Full reaction schemes used for the calculations are shown in Figure S1. The reaction conditions were assumed to be isothermal with temperature, *T* = 130 °C. The Δ*G̅*_f_^0^ values were calculated from the standard enthalpy and entropy of formation according to

$$\Delta {\mathrm{G̅}}_{f}^{o}=\Delta {\mathrm{H̅}}_{f}^{o}-T\Delta {\mathrm{S̅}}_{f}^{o}$$ 2

Here, *R* is the gas constant, Δ*S̅*_f_^*o*^ is the standard entropy of formation, Δ*H*_f_^*o*^ is the standard enthalpy of formation for each species, and standard pressure is 1 bar.

Thermodynamic properties in the gas phase, namely enthalpy (*H*), entropy (*S*), and Gibbs free energy (*G*), were obtained using the Group Additivity Values (GAV) method.^29^ This approach decomposes a molecule into its constituent functional groups, each associated with empirically derived contributions to the overall thermodynamic properties. The total property is obtained by summing the values for each group, along with corrections for interactions such as long-range effects when applicable. The GAV method is particularly effective for hydrocarbons and small organic molecules, offering a balance between computational efficiency and predictive accuracy. The implementation of the GAV method in the RMG software was used here.^30,31^ The method was benchmarked against high-level computations and the average error was found to be about 7.2 kJ/mol for enthalpy (Figures S2–S4).

### 2.9 Solubility Tests

To evaluate water solubility and pH resilience, samples were immersed for 1 h each in ultrapure water, followed by KOH solutions at pH 9.0 and pH 13.0 at room temperature. The pH was measured using a SevenCompact S220 pH meter (Mettler Toledo). After each one-hour immersion, samples were dried using a nitrogen blower and then oven-heated to 90 °C for 1 h. The thickness of dried samples was measured before and after each immersion step using spectroscopic ellipsometry. The tested samples include PVA films spin-coated on Si and modified with 10 cycles of IC-MPD, PDIC-MPD or TMA-H_2_O with short (≤1 s) exposure times (Table S5). A 53 nm PVA film with no modification served as a control as well as a 31 nm PVA film which was heated at the same temperature, pressure and duration as chemically modifed samples.

## 3 Results and Discussion

### 3.1 Molecular Layer Deposition on Nonabsorbing Substrates

Thin film growth via MLD was confirmed and characterized on nonabsorbing substrates (Figure 1b) for IC-MPD, PDIC-MPD, and TA-MPD chemistries. On silicon substrates, each chemistry exhibited a net thickness increase following each precursor dose, corresponding to the addition of molecular fragments at each step (Figure 2a). All chemistries demonstrated linear growth after an initialization period of approximately fifteen cycles. The steady state growth per cycle (GPC) of each chemistry was 0.14–0.19 Å/cycle.

**Figure 2:**
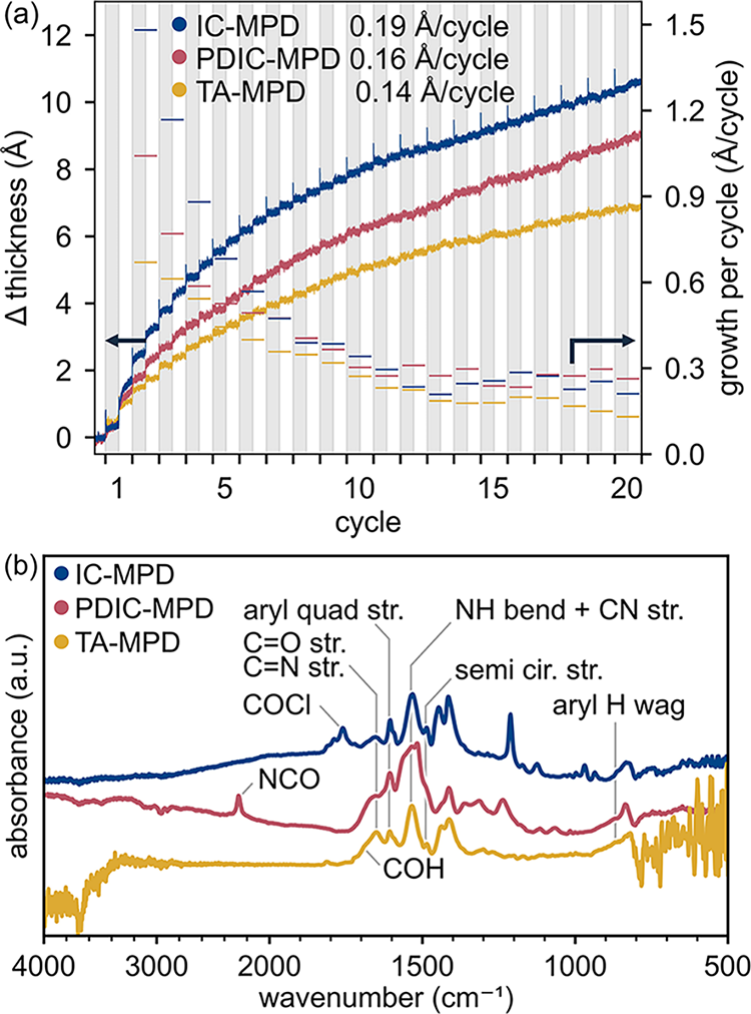
MLD of IC-MPD, PDIC-MPD, and TA-MPD on nonabsorbing substrates: (a) *In situ* ellipsometry measurements of MLD growth on Si substrates. Data corresponding with the right axis are three-point moving averages of growth per cycle (GPC). GPCs listed in the legend were determined from the average slopes past 15 cycles. The time span of each cycle was normalized to represent individual cycles on the abscissa. Gray bands correspond to electrophile steps and white bands to MPD steps. (b) FTIR difference spectra of MLD films grown on ZrO_2_ powder. These difference spectra were obtained by subtracting the measurements taken before MLD from those taken after.

During the initial cycles, the growth rates were higher than the steady state GPC, with distinct thickness increases after each precursor exposure. This behavior is likely due to an abundance of surface functional groups provided by the silicon substrate that supported high growth rates. Reduction of the GPC can be attributed to precursors which react with two surface functional groups, rather than one. Such “double reaction” events are known to reduce the number of substrate functional groups available for reaction in subsequent exposures, thereby lowering the GPC.^32^ After fifteen cycles, the GPC became linear, suggesting that the rate of double reactions had become negligible due to the sparse quantity of remaining functional groups.^33^

Mi et al.^10^ also reported growth of TA-MPD films for the fabrication of nanofiltration membranes. They observed a high GPC exceeding 6 Å/cycle at 100 °C using long dose times of 12–15 s. This GPC is high compared to this work and other MLD chemistries involving precursors of similar size, potentially indicating incomplete purging.^33^

We confirmed the formation of MLD polymers through FTIR analysis. Figure 2b shows measurements of ZrO_2_ powder substrates subtracted from the same sample after 3 MLD cycles. The difference spectra showed peaks at key positions indicative of polymer growth as well as unreacted precursors. Peak assignments are summarized in Table 1.

**Table 1:** Summary of FTIR peaks for MLD Films

chemistry	moiety location	IR interaction	wavenumber – cm^‑1^
IC-MPD	bulk film	amide I mode	1652
		amide II mode	1532
		amide III mode	1310
	substrate interface	ester C=O stretch	1760
		C-C-O stretch	1212
PDIC-MPD	bulk film	overtone of 1517 cm^–1^	3018
		urea C=O stretch	1660
		N-H in-plane bend + C-N stretch	1517
	substrate interface	urethane C=O stretch	1705
	unreacted functional groups	NCO asymmetric stretch	2274
TA-MPD	bulk film	C=N stretch	1652
	unreacted functional groups	aldehyde C=O stretch	1693
all	bulk film (shared aromatic moieties)	aryl quad stretch	1606
		semicircle stretch	1486

The IC-MPD spectra exhibited amide I (1652 cm^–1^) and amide II (1532 cm^–1^) modes, with a weak amide III peak at 1310 cm^–1^, confirming the amide reaction between IC and MPD. Ester reactions between the ZrO_2_ and IC were evident from C=O stretching at 1760 cm^–1^ and C-C-O stretching at 1212 cm^–1^. The 1760 cm^–1^ peak could also indicate the presence of unreacted acyl chloride groups.^34^ A derivative-shaped band appeared at 833 cm^–1^.

The PDIC-MPD spectra evidenced urea moieties through C=O stretching at 1660 cm^–1^ and N-H in-plane bending and C-N stretching at 1517 cm^–1^, with a weak overtone observed at 3018 cm^–1^.^34,35^ Urethane reactions between the zirconia and PDIC were difficult to detect, through a weak shoulder at 1705 cm^–1^ corresponded to urethane C=O stretching. Strong signals at 2274 cm^–1^ indicated unreacted isocyanate groups.^34^ A derivative-shaped band appeared at 835 cm^–1^.

The TA-MPD imine reaction was confirmed by C=N stretching at 1652 cm^–1^.^36^ Although aldehyde-amine reactions in solution are typically acid-catalyzed, this and other studies have demonstrated that these reactions can occur at the vapor-surface interface via MLD without the need for a catalysts.^36,37^ The shoulder at 1693 cm^–1^ corresponded to unreacted aldehyde C=O stretching.^34^ Incomplete reactions were evident for both PDIC-MPD and TA-MPD processes, suggesting subsaturation. This could result from either slow reaction rates or insufficient precursor supply due to low dosing pressures.

Thermodynamic calculations showed that at 130 °C, all MLD reactions are exothermic and exergonic, with negative Δ*H̅*_rxn_^o^ and Δ*G̅*_rxn_^o^ values (Table 2). The IC-MPD and TA-MPD reactions involve no net change in vapor species, resulting in a relatively low |*T*Δ*S̅*_rxn_^o^|, hence for these two reactions the Gibbs free energies are primarily determined by the corresponding Δ*H̅*_rxn_^o^ values.

**Table 2:** Thermodynamic Values for Reactions at 130°C^a^

reactants	Δ*G̅*_rxn_^o^ (kJ/mol)	Δ*H̅*_rxn_^o^ (kJ/mol)	Δ*S̅*_rxn_^o^ (J/mol)
IC-MPD	–69.0	–83.8	–36.6
PDIC-MPD	–36.7	–130.1	–231.7
TA-MPD	–19.6	–19.8	–0.4

a The values reported here are based on enthalpies and entropies of formation for a single reaction between the electrophile and MPD.

### 3.2 Material Growth on Polymer Substrates

With MLD characterized on nonabsorbing substrates, we turn to investigate the three chemistries on polymeric substrates, including spin-coated PVA, PMMA, and PS films. Unlike the silicon and ZrO_2_ substrates in the previous section, the polymeric substrates can absorb MLD precursors, potentially leading to subsurface organic VPI growth (Figure 1c). Thickness changes were monitored *in situ with* ellipsometry during moderate precursor dose (5 s) and purge (325 s) steps over ten cycles.

PVA exhibited a thickness increase in response to acyl chloride (IC) and isocyanate (PDIC) chemistries (Figure 3a). Over the ten cycles of PDIC-MPD, the PVA substrate exhibited a small, constant thickness change of ∼0.8 Å per cycle. IC-MPD, in contrast, caused an exceptionally large thickness increase of 22 Å in PVA on the first cycle, followed by diminishing growth in subsequent cycles. During the process, the changes in sample thickness could be attributed to a combination of surface film growth, swelling, and material growth within the polymer substrate volume.

**Figure 3:**
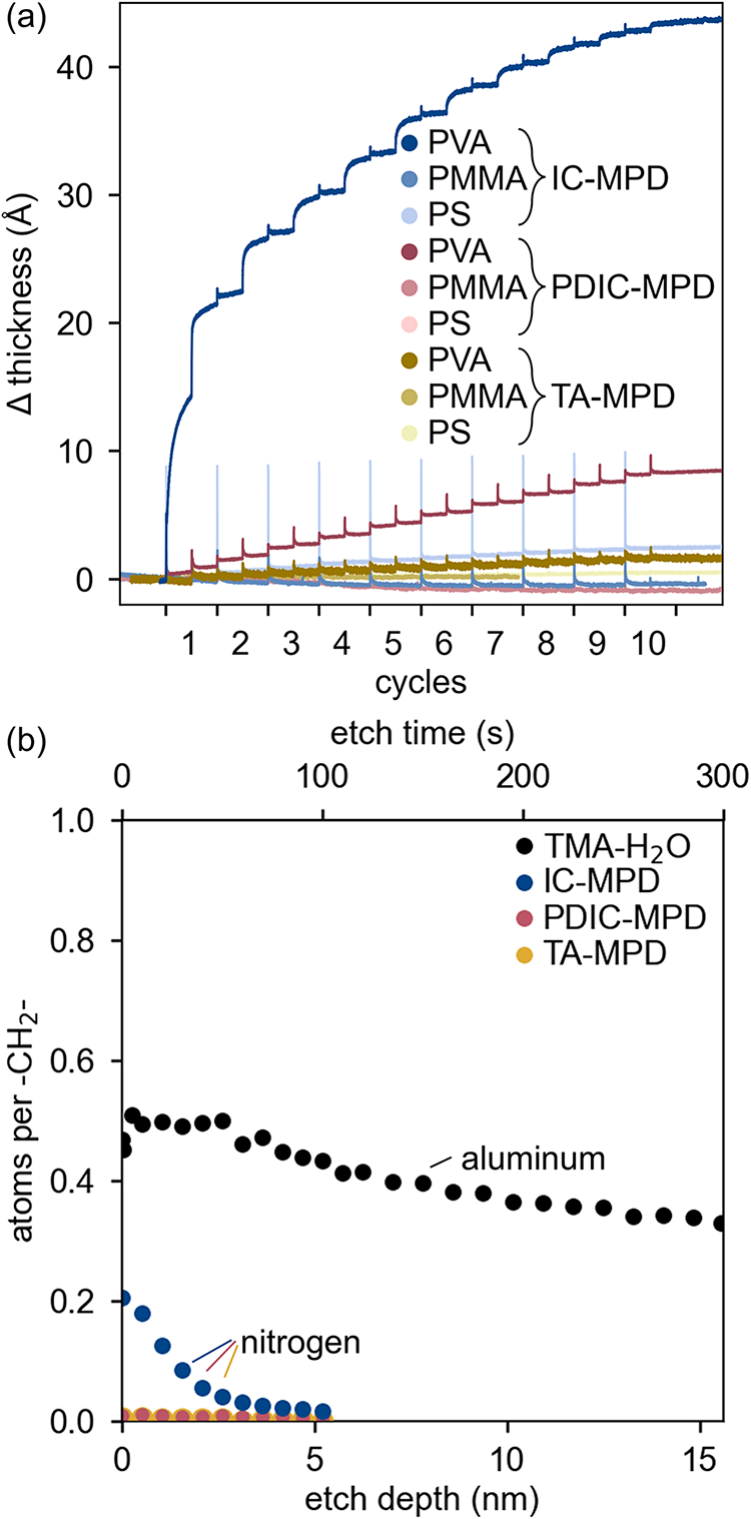
(a) Thickness change of polymer substrates over 10 cycles (5 s doses/325 s purges), measured in situ using ellipsometry. The initial film thicknesses were ∼50 nm for PMMA, ∼65 nm for PS, and ∼85 nm for PVA, as listed in Table S3. The time span of each cycle was normalized to represent individual cycles on the abscissa. Electrophiles (IC, PDIC, and TA) were introduced at the start of each cycle, with MPD dosed at the second half. (b) XPS depth profiles of PVA samples from (a) along with a PVA sample exposed to 10 cycles of TMA-H_2_O (0.3 s doses/330 s purges). Atomic concentrations of key elements (aluminum and nitrogen) were normalized to the −CH_2_– concentration (deconvoluted from the C 1s signal), which was present only in the PVA. XPS survey (Figure S5) and deconvolution (Figure S6) data for PVA are found in the SI, Section II. Etch depth was estimated from an etch rate of 0.052 nm/s, detailed in the SI, Section III.

Besides these two examples, all other MLD-substrate combinations yielded negligible 10-cycle thickness changes of <0.25 nm (<0.4% of their overall thickness, listed in Table S3). Further evidence of nongrowth was provided by XPS measurements of the PS and PMMA samples which showed negligible nitrogen 1s signals within the top ∼5 nm (100 s of etching), as shown in Figure S7.

VPI requires entrapment through chemical reaction or a physical process, neither of which was detected in these cases.^1^ For the PS samples, the lack of reactive functional groups can explain the nongrowth. The same can be said for PMMA, which has otherwise served as a model substrate for inorganic VPI. While its methacrylate groups serve as suitable Lewis bases for reaction with metal–organic, Lewis acid precursors, they are poor nucleophiles and fail to interact with organic electrophiles.

Despite the lack of net growth, sharp peaks in the thickness data appeared at each dose in Figure 3a. These temporal spikes in the thickness data can be attributed to transient adsorption/desorption of precursors, which may have affected the measurement by altering the polymer optical properties or directly increasing the polymer thickness through swelling or thermal expansion arising from temperature differences between the precursor and substrate.^38,39^

To better understand VPI material growth within PVA, we performed XPS depth profiling measurements of the three organic chemistries, shown in Figure 3b. For a comparison to inorganic VPI, we also measured a PVA sample modified with 10 cycles of a TMA-H_2_O chemistry (10 cycles, 0.3 s doses/330 s purges). Aluminum (from TMA) and nitrogen (from the organic species) served as indicators of precursor incorporation into the PVA. The data were normalized to the signal of single bonded C-C atoms found in the vinyl backbone of PVA which can be assumed to remain constant throughout the sample thickness. The etch depth was approximated as 5.2 nm per 100 s of etching, as detailed in the SI, Section IV.

The XPS analysis showed that TMA and H_2_O infiltrated and reacted throughout the sample depth, while the organic chemistries exhibited shallow, if any, infiltration. Although *in situ* ellipsometry detected deposition for PDIC-MPD, the XPS nitrogen 1s signal was on the same scale as the instrument noise. Similarly, TA-MPD produced negligible nitrogen 1s signals at all depths. In contrast, the nitrogen 1s concentration for IC-MPD decreased from ∼10% at the surface to 0% within the first ∼5 nm.

Overall, these results suggest that the organic precursors diffused into and reacted with PVA at a significantly lower rate than TMA/H_2_O. Additionally, TA and PDIC exhibited slower rates of reaction with substrate functional groups compared to IC. These observations raise important questions: What are the precise reaction and diffusion rates of these precursors in PVA? Could PDIC incorporation be enhanced with longer exposure times?

### 3.3 Quantification of Vapor Phase Infiltration

The ability to deposit materials by VPI within polymer substrates depends on the initial reactions with that substrate, i.e., nucleation of the growth. In the case of PVA, the electrophiles would react with hydroxyl pendant groups, as depicted in Figure 4.

**Figure 4:**
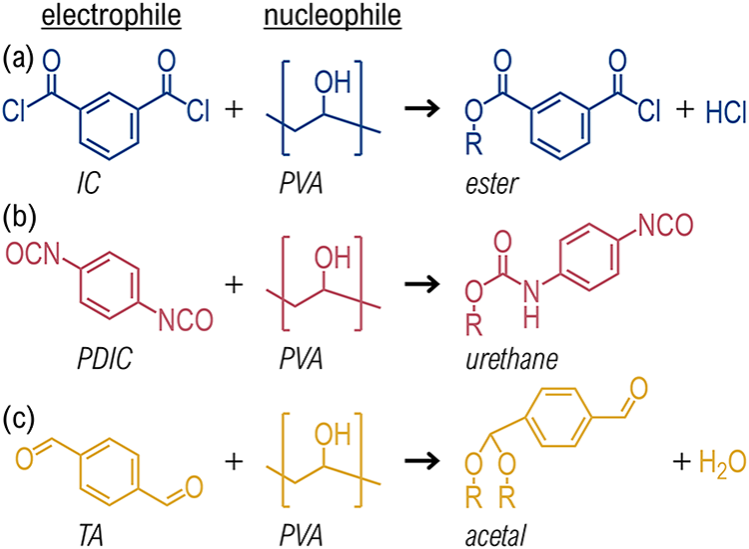
Possible reactions between MLD electrophile precursors and PVA substrate hydroxyls. (a) Ester condensation. (b) Urethane addition. (c) Acetal condensation.

Before discussing the kinetics of these reactions, we first examine their thermodynamics, summarized in Table 3. The IC-PVA and PDIC-PVA reactions exhibited negative Δ*G*_rxn_^o^ values of lower magnitude than those involving MPD. Both PDIC-PVA and TA-PVA reactions were entropy-driven (|Δ*H*| ≪ |*T*Δ*S*|), which, in combination with only a moderately exothermic enthalpy, made the TA-PVA reaction thermodynamically neutral, with Δ*G*_rxn_^o^ close to zero within the uncertainty. Thus, the lack of observed TA-MPD growth in PVA in Figure 3a is attributed to thermodynamic limitations of TA-PVA nucleation. To be thorough, we also performed calculations for the semistable hemiacetal intermediate which had a larger, positive Δ*G*_rxn_^o^ than the TA-PVA reaction that produced an acetal.

**Table 3:** Thermodynamic Values for Reactions between the Electrophile Vapors and PVA^a^ at 130 °C

reactants	Δ*G̅*_rxn_^o^ – kJ/mol	Δ*H̅*_rxn_^o^ – kJ/mol	Δ*S̅*_rxn_^o^ – J/mol
IC-PVA	–9.2	–7.3	4.8
PDIC-PVA	–11.8	–92.9	–201.2
TA-PVA	2.3	–42.3	–110.8
TA-PVA (hemiacetal product)	19.5	–36.4	–138.6

a Corresponding reaction schemes are shown in Figure S1.

Transport and reaction kinetics of VPI during the initial organic precursor exposures were quantified by fitting QCM mass uptake data to the Ren-McGuinness reaction-diffusion transport model.^24^ This model incorporates three key components 1. Fickian diffusion, 2. reaction kinetics, and 3. diffusion hindrance resulting from precursors that have reacted with the substrate. The model examines a single precursor exposure on a pristine substrate, thereby describing the nucleation reactions of VPI. The mathematical details, execution methods, assumptions, and nuances of the model used in this study are provided in the SI, Section V.

Ren et al. developed this model and used it to predict the outcomes for PMMA exposed to TMA.^24^ Here, we used this model to describe the behavior of the VPI chemistries in PVA and provide a quantitative understanding of the nucleation process for each unique precursor-substrate system. By fitting the experimental QCM data to this model, we estimated the reaction rate (*k*), initial diffusion coefficient (*D*_0_), and hindering factor (*K*′) for the interaction between the electrophile precursors and the PVA substrate. These parameters can fully describe the VPI system in three dimensionless numbers: the Damköhler number (*Da*), diffusion hindrance (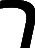), and concentration ratio (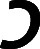).^6^ 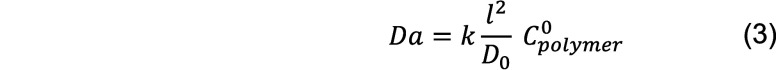 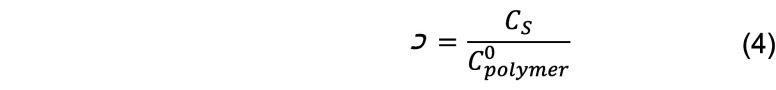 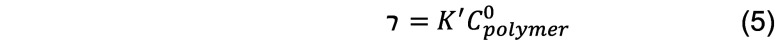
Here, *Da* represents the ratio between the rate of diffusion within the polymer substrate (of film thickness *l*) and precursor-polymer reaction. A low *Da* indicates rapid diffusion relative to reaction (reaction-limited), while a high *Da* indicates a diffusion-limited system. The concentration parameter, 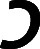 is the ratio between dose concentration (*C*_S_) and the density of accessible functional groups in the substrate (*C*_polymer_^0^).^6^ Note that *C*_polymer_^0^ is not equal to total molar concentration of functional groups, but rather the amount of groups available for reaction which may be reduced due to steric hindrance or from double reactions with the precursor. The hindrance parameter, 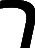 quantifies the extent to which diffusivity is locally reduced once a precursor has reacted with the substrate.

QCM data were fit to the model to estimate the VPI parameters for the organic IC-PVA and PDIC-PVA, and inorganic TMA-PVA systems (Figure 5). The results, summarized in Table 4, are compared with those for TMA-PMMA from Ren et al.^24^ Ren et al used a “subjective” fit of *k* from a single, long exposure experiment. In contrast, our method utilized both short and long exposure experiments for an objective fit of *k*. Our study also used different experimental conditions including thinner spin-coated films, lower dose pressures, and shorter exposure/purge times. The lower dose pressures led to comparatively lower 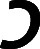 values, while thinner films facilitated faster saturation, allowing for shorter exposure and purge times. Despite these differences, *C*_polymer_^0^ remained within the same order of magnitude across all systems, assuring the comparability of both methods.

**Figure 5:**
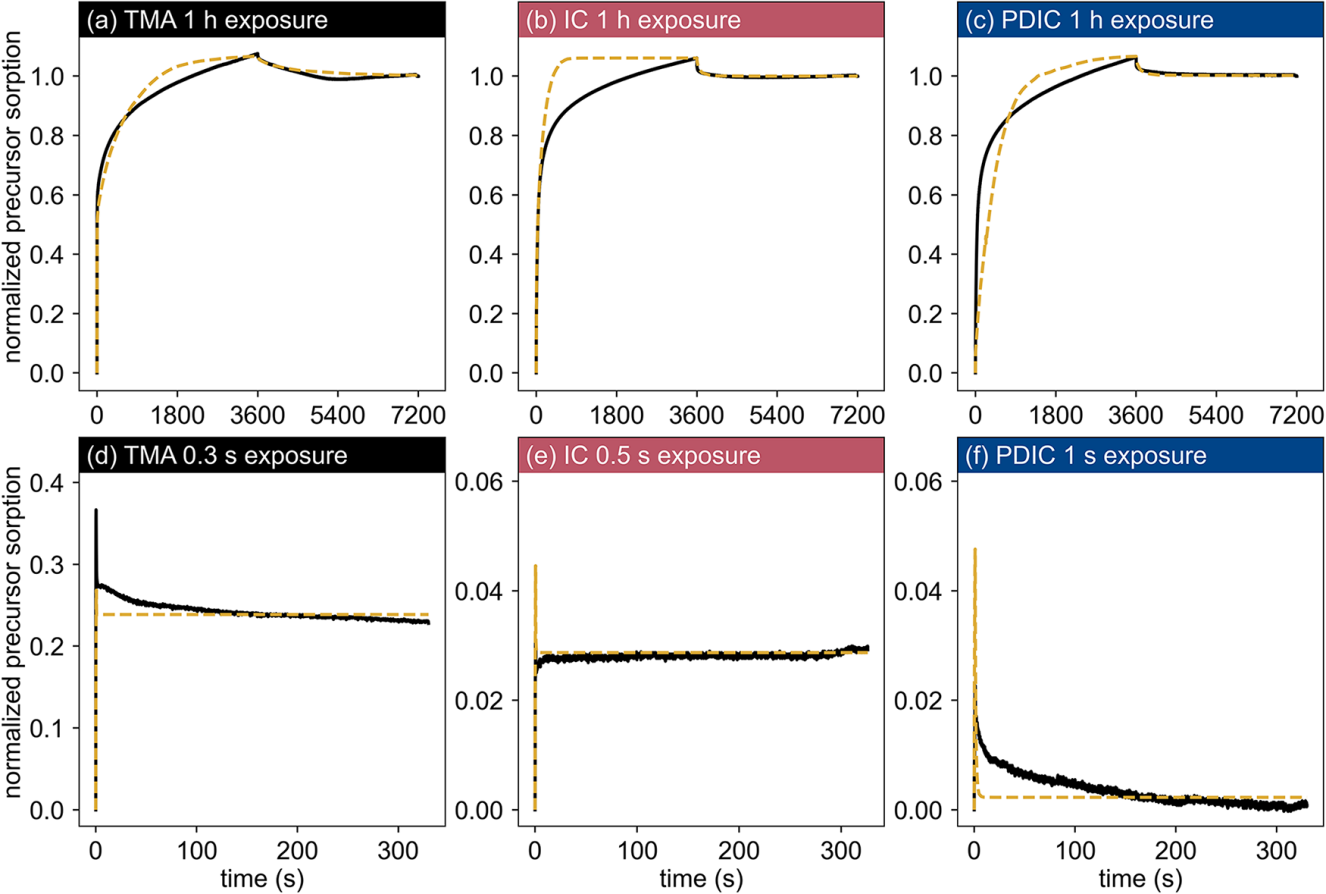
Kinetics of precursors in PVA as measured by QCM. Plots (a–c) show data for long precursor exposures on PVA, normalized to their final measurement. Plots (d–f) show data for short precursor exposures on PVA, normalized to the final measurement of the corresponding reaction data in (a–c). Dotted lines show the Ren-McGuinness model with parameters listed in Table 4. Shared axis labels are shown on the leftmost and bottommost plots.

**Table 4:** VPI Parameters Fit by the Ren-McGuinness Model

precursor-substrate	*K*’ – cm^3^/mol	*k* – cm^3^/mol	*D*_0_ – cm^2^/s	*C^0^*_polymer_ – mol/cm^3^	*C*_s_ – mol/cm^3^	*l* – nm	*Da*	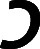	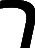
IC-PVA	3000	2000	1 × 10^–11^	2 × 10^–3^	7 × 10^–5^	39	6	0.04	5
PDIC-PVA	4000	30	1 × 10^–11^	1 × 10^–3^	9 × 10^–5^	42	0.07	0.06	5
TMA-PVA	3000	3000	5 × 10^–10^	4 × 10^–3^	2 × 10^–4^	36	0.3	0.06	10
TMA-PMMA^a^	670	0.6	1.65 × 10^–10^	9.7 × 10^–3^	4.4 × 10^–3^	480	0.08	0.45	6.5

a These results of this row originate for ref 24 but differ from the original work due to a correction that accounts for the net mass change of a reaction. Additional details about this correction can be found in the SI, Section V.

#### 3.3.1 Comparison of Damköhler Numbers

The value of *D*_0_ for organic precursors in PVA were over an order of magnitude lower than that of TMA, likely due to the bulkier and more rigid structures of the organic precursors. While the *D*_0_ values of PDIC and IC were close in value, *k* of IC was 2 orders of magnitude higher than that of PDIC. Consequently, the rapid ester reaction in the IC-PVA system was diffusion-limited (*Da* > 1), whereas the slower urethane reaction in the PDIC-PVA was reaction-limited (*Da* < 1).

Despite the substantial differences in *Da*, both reaction-limited (PDIC) and diffusion-limited (IC) systems exhibited similar mass uptake during extended static exposures (Figure 5b,c) in terms of the profile and value. Absolute mass uptake data are shown in Figure S10. The effects of *Da* differences were primarily evident in the experiments with short exposures. For reaction-limited PDIC, short precursor doses resulted in a minimal net mass gain due to insufficient reaction time (Figure 5f). In contrast, a short dose of diffusion-limited IC resulted in immediate and permanent mass uptake with negligible desorption of unreacted precursor (Figure 5e). These findings underscore the importance of using both long and short exposures to fit the rate of reaction.

In general, TMA is known to exhibit lower reactivity in PMMA compared to other polymers (e.g., poly­(ethylene 4 terephthalate glycol) (PET-G), polycaprolactone (PCL), poly (lactic acid) (PLA) and poly­(butylene succinate) (PBS)),^40,41^ and this was observed in the QCM measurements. In PMMA, TMA exhibited reaction-limited behavior (*Da* < 1) due to its relatively low *k* and relatively high *D*_0_. TMA demonstrated much greater *D*_0_ and *k* in PVA with *Da* much closer to unity, but still less than 1. The film thickness in the PMMA experiment was significantly larger than the PVA experiments; if the PVA and PMMA film thicknesses were equal, the difference in *Da* would be even greater, per eq 3.

#### 3.3.2 Comparison of *C*_polymer_^0^

The *C*_polymer_^0^ values of PVA are lower when using PDIC and IC compared to TMA which indicates that functional groups were less accessible to the organic molecules on account of more steric hindrance or double reactions. Additionally, because ∼12% of the PVA functional groups were acetates, TMA had more functional groups available for reaction since TMA can react with esters while IC and PDIC cannot.^42^

Although PMMA and PVA have similar mass densities, the molecular weight of a PMMA repeat unit is nearly twice that of PVA. This might suggest that PMMA has fewer accessible functional groups than PVA. However, we found that *C*_polymer_^0^ for PMMA was higher than for PVA when using TMA. We propose that in PVA, a single TMA is more likely to react with multiple functional groups (double or triple reactions) due to the closer proximity of reactive sites and higher reaction rates compared to PMMA.^33^ More reactions per precursor molecule on average would reduce the number of available functional groups for unreacted TMA. Additionally, each reaction between TMA and a hydroxyl results in mass loss through methane byproduct formation. If multiple reactions occur per TMA, the net mass gain per reacted TMA is lower than estimated in our model, leading to an underestimation of *C*_polymer_^0^.

#### Comparison of  
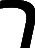


3.3.3

Reactions of IC and PDIC in PVA hindered diffusion to a similar extent, as indicated by comparable 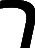 values. This may be attributed to their structural similarity. TMA exhibited a higher 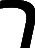 than either organic precursor, likely due to multiple reactions and its ability to react with acetate groups in PVA. Compared to PMMA, TMA showed a higher 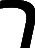 in PVA, showing that a given precursor does not necessarily exhibit similar hindrance effects across different substrates. Again, more reactions per TMA molecule may explain this difference.

These findings explain the minimal thickness change observed with short PDIC-MPD pulses on PVA (Figure 3a). Although PDIC’s reaction with the substrate was thermodynamically favorable, its low reaction rate (*k*) and diffusivity (*D*_0_) led to minimal material deposition during dosing. Subsequent MPD doses, which cannot react directly with the PVA, encountered few available isocyanate groups. In contrast, IC-MPD and TMA-H_2_O experiments exhibited rapid and extensive initial reactions with the substrate generating a higher density of functional groups available for subsequent precursor exposures.

### 3.4 Dissolution and pH Resistance

We hypothesized that samples modified with organic chemistries would resist dissolution in aqueous solutions, whereas those modified with TMA/H_2_O would dissolve due to hydrolysis of the resulting aluminum alkoxide (Al-O-C bond).^43^ To test this, we prepared PVA films modified with 10 cycles of short precursor exposures (≤1 s; Table S5) using IC–MPD, PDIC–MPD, or TMA–H_2_O. Control samples were also prepared – an unmodified PVA film as well as a “heated PVA” film exposed to the same pressure, temperature and duration as chemically modified samples. The films were then immersed for one hour each in ultrapure water, followed by KOH solutions at pH 9 and 13. The samples were dried after each immersion step to measure the thickness changes as shown in Figure 6. Absolute thickness measurements are reported in Table S7.

**Figure 6:**
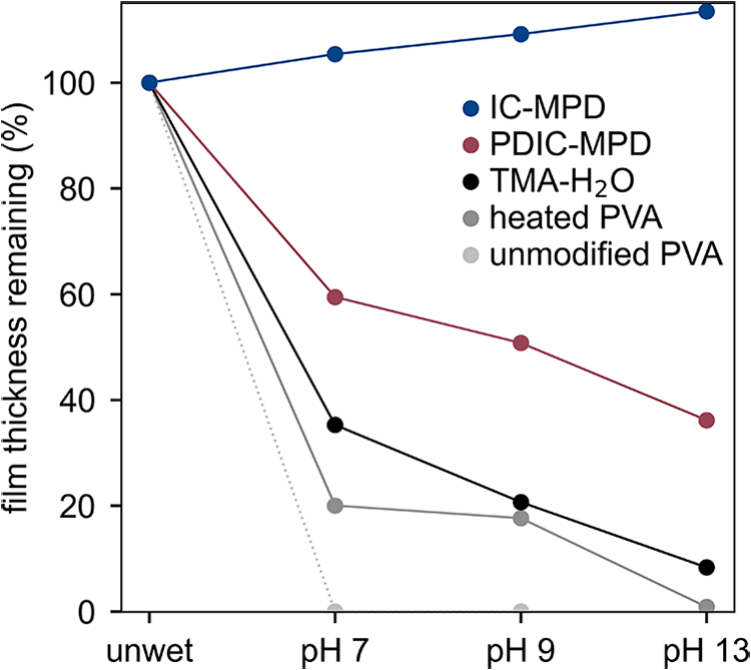
Film thickness of modified PVA after sequential immersion in baths of increasing pH, used to assess resistance to dissolution. Samples were modified with 10 cycles of their respective chemistry using short (≤1 s) exposures. “Heated PVA” resided in the reaction chamber at the same temperature, pressure, and duration as chemically modified samples. Samples were dried between baths and thickness was measured using ellipsometry.

While the unmodified PVA dissolved in pure water, the heated PVA retained a fraction of its thickness until immersion in the pH 13 bath. This points to heat-induced cross-linking of the film.^22^ Likewise, PVA films modified with PDIC-MPD and TMA-H_2_O progressively decreased in thickness with each subsequent bath. In contrast, PVA modified with IC-MPD retained the original film thickness, which even increased slightly after each subsequent immersion demonstrating excellent dissolution resistance up to pH 13. These results show that IC-MPD treatment, even using short (≤1 s) exposures, can preserve the film structure and prevent dissolution.

The relative effectiveness of PDIC-MPD vs IC-MPD could be correlated to mass uptake. QCM measurements were taken during the preparation of these samples and show that the mass uptake of PDIC-MPD was an order of magnitude lower than that of IC-MPD (Figure S11).

The TMA-H_2_O modified PVA experienced only a marginally improved degree of thickness retention compared to heated PVA despite exhibiting the greatest mass uptake (Figure S11). After the pH 13 bath, only 11% of the film thickness remained, as the Al-O-C bonds formed between PVA and TMA were susceptible to hydrolysis, even at neutral pH.^2,44,45^ Although vulnerable to water, TMA-H_2_O treatment is known to provide polymers with protection against dissolution in organic solvents via two mechanisms: cross-linking and diffusion barrier formation.^18^ Sasson et al. found that the diffusion barrier mechanism – where material deposition inhibits solvent uptake within the polymer – was likely the more effective phenomenon to prevent dissolution of alumina-modified P­(S-*r*-MMA) in toluene.^19^

Compared to oxides, polymeric materials generally serve as poor diffusion barriers. Therefore, we can attribute cross-linking as the mechanism of dissolution protection if a significant amount of cross-linking (rather than propagation of the IC-MPD chain) can be quantified. To do this, we compared the relative mass uptake between electrophiles and MPD with QCM measurements during vapor modification. If the ratio of total molar increase during electrophile doses was equal to the increase during MPD doses, then material deposition primarily occurred at the functional end groups of the preceding precursor, rather than forming cross-links. Conversely, if the electrophile increased more than MPD, it would indicate that some fraction of the electrophiles had reacted twice to form cross-links rather than providing a functional group for the subsequent MPD dose. The QCM data (Table 5) indicate that the latter scenario was true for both IC-MPD and PDIC-MPD – nearly twice the molar amount of electrophile was incorporated into the PVA than MPD, indicating that the terminal end of each IC was equally likely to react with PVA as with a subsequent MPD. Details of the calculations of molar increase from QCM measurements are detailed in the SI, Section VI.

**Table 5:** Total Mass and Molar Gains for Ten Short Dose Cycles of Organic Chemistries in PVA

	total gains of first precursor		total gains of second precursor		precursor ratio
	ng/cm^2^	nmol/cm^2^	ng/cm^2^	nmol/cm^2^	mol E/mol N
IC-MPD	193	1.16	49	0.68	1.9
PDIC-MPD	15	0.096	5.4	0.050	1.9

These results demonstrate that organic precursors provide solvent protection through cross-linking while remaining resistant to hydrolysis, even at high pH. Treatment is more effective with systems exhibiting higher reactivity (IC vs PDIC) and can be achieved with very short pulses.

## 4 Conclusions

This study investigated the physical mechanisms of organic VPI and demonstrated its ability to enhance dissolution resistance in polymers. It established a quantitative framework to better understand the kinetics and thermodynamics of VPI processes but questions about the chemical mechanisms remain. While electron pushing mechanisms are well established for substitution reactions in solution stabilized contexts, future studies will need to explore the mechanisms of electron transfer in vapor-surface and polymer-absorbed environments.

As more organic and inorganic VPI systems are quantified, the accumulation of dimensionless parameters will allow for study and understanding structure-property relations between precursor-substrate systems and their diffusion–reaction behavior. This information will facilitate rational selection of precursor chemistries and optimization of VPI process variables such as temperature, pressure, and exposure time. It could allow researchers to predict the behavior of untested combinations, accelerating the design of chemically robust coatings, solvent-stable membranes, and functionalized soft materials with tailored subsurface properties. The implications extend beyond VPI into gas sorption where the reaction-diffusion behavior can similarly be quantified.

## Supplementary Material



## Notes

Tabulated experimental information; group additivity calculation basis; XPS depth profiles; calibration of XPS etch depth; details of the Ren-McGuinness reaction diffusion model; basis for calculation of double reactions from QCM results (PDF)

## References

[1] Leng C. Z., Losego M. D. (2017). Vapor Phase Infiltration (VPI) for Transforming Polymers
into Organic–Inorganic Hybrid Materials: A Critical Review
of Current Progress and Future Challenges. Mater.
Horiz..

[2] Choi D.-w., Yoo M., Lee H. M., Park J., Kim H. Y., Park J.-S. (2016). A Study
on the Growth Behavior and Stability of Molecular Layer Deposited
Alucone Films Using Diethylene Glycol and Trimethyl Aluminum Precursors,
and the Enhancement of Diffusion Barrier Properties by Atomic Layer
Deposited Al2O3 Capping. ACS Appl. Mater. Interfaces.

[3] Ko M., Kim H.-U., Jeon N. (2023). Sequential Infiltration Synthesis
with Organic Co-Reactants for Extensively Swollen Organic–Inorganic
Hybrid Thin Films. ACS Appl. Polym. Mater..

[4] Lee S.-M., Pippel E., Gösele U., Dresbach C., Qin Y., Chandran C. V., Bräuniger T., Hause G., Knez M. (2009). Greatly Increased
Toughness of Infiltrated Spider Silk. Science.

[5] Lee S.-M., Pippel E., Moutanabbir O., Gunkel I., Thurn-Albrecht T., Knez M. (2010). Improved Mechanical Stability of Dried Collagen Membrane after Metal
Infiltration. ACS Appl. Mater. Interfaces.

[6] Welch B. C., Casetta J., Pathak R., Elam J. W., Pochat-Bohatier C., Miele P., Segal-Peretz T. (2025). Atomic Layer
Deposition of Nanofilms
on Porous Polymer Substrates: Strategies for Success. J. Vac. Sci. Technol., A.

[7] Zara D. L., Zhang F., Sun F., Bailey M. R., Quayle M. J., Petersson G., Folestad S., van Ommen J. R. (2021). Drug Powders
with Tunable Wettability by Atomic and Molecular Layer Deposition:
From Highly Hydrophilic to Superhydrophobic. Appl. Mater. Today.

[8] Vasudevan S. A., Xu Y., Karwal S., van Ostaay H. G. M. E., Meesters G. M. H., Talebi M., Sudhölter E. J. R., van Ommen J. R. (2015). Controlled
Release from Protein Particles Encapsulated by Molecular Layer Deposition. Chem. Commun..

[9] Welch B. C., McIntee O. M., Myers T. J., Greenberg A. R., Bright V. M., George S. M. (2021). Molecular Layer Deposition for the
Fabrication of Desalination Membranes with Tunable Metrics. Desalination.

[10] Mi K., Xiong S., Lu Y., Wang Y. (2024). Precise Fabrication
of Robust Conjugated Microporous Polymer Membranes via Oxidative Molecular
Layer Deposition for Efficient Organic Solvent Nanofiltration. Chem. Mater..

[11] Xiong S., Sheng T., Kong L., Zhong Z., Huang J., Wang Y. (2016). Enhanced Performances
of Polypropylene Membranes by Molecular Layer
Deposition of Polyimide. Chin. J. Chem. Eng..

[12] Welch B. C., McIntee O. M., Ode A. B., McKenzie B. B., Greenberg A. R., Bright V. M., George S. M. (2020). Continuous Polymer Films Deposited
on Top of Porous Substrates Using Plasma-Enhanced Atomic Layer Deposition
and Molecular Layer Deposition. J. Vac. Sci.
Technol., A.

[13] Mehregan M., Stalla D., Luebbert G., Baratta L., Brathwaite K. G., Wyatt Q. K., Paranamana N. C., Young M. J. (2023). Compressible Sponge
Electrodes by Oxidative Molecular Layer Deposition (oMLD) of Polyethylenedioxythiophene
(PEDOT) onto Open-Cell Polyurethane Sponges. Nanotechnology.

[14] George S. M. (2010). Atomic
Layer Deposition: An Overview. Chem. Rev..

[15] Meng X. (2017). An Overview
of Molecular Layer Deposition for Organic and Organic–Inorganic
Hybrid Materials: Mechanisms, Growth Characteristics, and Promising
Applications. J. Mater. Chem. A.

[16] Wilson C.
A., Grubbs R. K., George S. M. (2005). Nucleation and Growth during Al2O3
Atomic Layer Deposition on Polymers. Chem. Mater..

[17] Peng Q., Tseng Y.-C., Darling S. B., Elam J. W. (2010). Nanoscopic Patterned
Materials with Tunable Dimensions via Atomic Layer Deposition on Block
Copolymers. Adv. Mater..

[18] McGuinness E. K., Leng C. Z., Losego M. D. (2020). Increased
Chemical Stability of Vapor-Phase
Infiltrated AlOx–Poly­(Methyl Methacrylate) Hybrid Materials. ACS Appl. Polym. Mater..

[19] Sasson G., Welch B. C., Zhang H., Diesendruck C. E., Segal-Peretz T. (2025). Enhancing the Solvent Resistance
of Random Copolymer
Films via Sequential Infiltration Synthesis: Low Functional Group
Density Can Make a Large Impact. Polym. Adv.
Technol..

[20] McGuinness E. K., Zhang F., Ma Y., Lively R. P., Losego M. D. (2019). Vapor Phase
Infiltration of Metal Oxides into Nanoporous Polymers for Organic
Solvent Separation Membranes. Chem. Mater..

[21] Park S. W., Bae K., Kim J. W., Lee G. B., Choi B.-H., Lee M. H., Shim J. H. (2016). Chemical Protection of Polycarbonate Surfaces by Atomic
Layer Deposition of Alumina with Oxygen Plasma Pretreatment. Adv. Mater. Interfaces.

[22] Sau S., Pandit S., Kundu S. (2021). Crosslinked
Poly (Vinyl Alcohol):
Structural, Optical and Mechanical Properties. Surf. Interfaces.

[23] Giménez V., Mantecón A., Cádiz V. (1996). Modification
of Poly­(Vinyl Alcohol)
with Acid Chlorides and Crosslinking with Difunctional Hardeners. J. Polym. Sci., Part A:Polym. Chem..

[24] Ren Y., McGuinness E. K., Huang C., Joseph V. R., Lively R. P., Losego M. D. (2021). Reaction–Diffusion
Transport Model to Predict
Precursor Uptake and Spatial Distribution in Vapor-Phase Infiltration
Processes. Chem. Mater..

[25] Elam J. W., Groner M. D., George S. M. (2002). Viscous
Flow Reactor with Quartz
Crystal Microbalance for Thin Film Growth by Atomic Layer Deposition. Rev. Sci. Instrum..

[26] Myers T. J., George S. M. (2021). Molecular Layer
Deposition of Nylon 2,6 Polyamide Polymer
on Flat and Particle Substrates in an Isothermal Enclosure Containing
a Rotary Reactor. J. Vac. Sci. Technol., A.

[27] Higgs D. J., DuMont J. W., Sharma K., George S. M. (2018). Spatial Molecular
Layer Deposition of Polyamide Thin Films on Flexible Polymer Substrates
Using a Rotating Cylinder Reactor. J. Vac. Sci.
Technol., A.

[28] Arnau, A. ; Soares, D. Fundamentals of Piezoelectricity. In Piezoelectric Transducers and Applications; Springer: Berlin, Heidelberg, 2009; pp 1–38 10.1007/978-3-540-77508-9_1.

[29] Benson S. W., Cruickshank F. R., Golden D. M., Haugen G. R., O’Neal H. E., Rodgers A. S., Shaw R., Walsh R. (1969). Additivity Rules for
the Estimation of Thermochemical Properties. Chem. Rev..

[30] Johnson M. S., Dong X., Grinberg Dana A., Chung Y., Farina D., Gillis R. J., Liu M., Yee N. W., Blondal K., Mazeau E., Grambow C. A., Payne A. M., Spiekermann K. A., Pang H.-W., Goldsmith C. F., West R. H., Green W. H. (2022). RMG Database for Chemical Property
Prediction. J. Chem. Inf. Model..

[31] Liu M., Grinberg
Dana A., Johnson M. S., Goldman M. J., Jocher A., Payne A. M., Grambow C. A., Han K., Yee N. W., Mazeau E. J., Blondal K., West R. H., Goldsmith C. F., Green W. H. (2021). Reaction Mechanism Generator v3.0: Advances in Automatic
Mechanism Generation. J. Chem. Inf. Model..

[32] Adamczyk N. M., Dameron A. A., George S. M. (2008). Molecular Layer Deposition of Poly­(p-Phenylene
Terephthalamide) Films Using Terephthaloyl Chloride and p-Phenylenediamine. Langmuir.

[33] Welch B. C., Antonio E. N., Chaney T. P., McIntee O. M., Strzalka J., Bright V. M., Greenberg A. R., Segal-Peretz T., Toney M., George S. M. (2024). Building Semipermeable
Films One
Monomer at a Time: Structural Advantages via Molecular Layer Deposition
vs Interfacial Polymerization. Chem. Mater..

[34] Larkin, P. J. Chapter 6 - IR and Raman Spectra–Structure Correlations: Characteristic Group Frequencies. In Infrared and Raman Spectroscopy, Second ed.; Larkin, P. J. , Ed.; Elsevier, 2018; pp 85–134 10.1016/B978-0-12-804162-8.00006-9.

[35] Kim A., Filler M. A., Kim S., Bent S. F. (2005). Layer-by-Layer Growth
on Ge(100) via Spontaneous Urea Coupling Reactions. J. Am. Chem. Soc..

[36] Mi K., Ji X., Xiong S., Wang Y. (2024). Molecular Layer Deposition of Conjugated
Microporous Polymers (CMPs) Thin Films for Fast Molecular Sieving. Sep. Purif. Technol..

[37] Yoshimura T., Ito S., Nakayama T., Matsumoto K. (2007). Orientation-Controlled
Molecule-by-Molecule
Polymer Wire Growth by the Carrier-Gas-Type Organic Chemical Vapor
Deposition and the Molecular Layer Deposition. Appl. Phys. Lett..

[38] Petit R. R., Li J., Van de Voorde B., Van Vlierberghe S., Smet P. F., Detavernier C. (2021). Atomic Layer Deposition on Polymer
Thin Films: On the Role of Precursor Infiltration and Reactivity. ACS Appl. Mater. Interfaces.

[39] Pathak R., Rozyyev V., Shevate R., Mane A. U., Elam J. W. (2023). Controlling
Nanoscale Pore Size and Wall Composition in Polycarbonate Membranes
via Atomic Layer Deposition and Sequential Infiltration Synthesis:
Implications for High Water Permeance. ACS Appl.
Nano Mater..

[40] Parsons G. N., Atanasov S. E., Dandley E. C., Devine C. K., Gong B., Jur J. S., Lee K., Oldham C. J., Peng Q., Spagnola J. C., Williams P. S. (2013). Mechanisms and Reactions during Atomic
Layer Deposition on Polymers. Coord. Chem. Rev..

[41] Perego, M. ; Motta, A. ; Ronnby, K. ; Yap, F. T. J. ; Seguini, G. ; Wiemer, C. ; Nolan, M. On the Differences in Trimethylaluminum Infiltration into PMMA and PLA Polymers for Sequential Infiltration Synthesis: Insights from Experiment and First Principles Simulations ChemRxiv 2024 10.26434/chemrxiv-2024-zdk5k. PMC12481621 41036102

[42] Gong B., Parsons G. N. (2012). Quantitative in
Situ Infrared Analysis of Reactions
between Trimethylaluminum and Polymers during Al2O3 Atomic Layer Deposition. J. Mater. Chem..

[43] Wengrovius J. H., Garbauskas M. F., Williams E. A., Goint R. C., Donahue P. E., Smith J. F. (1986). Aluminum
Alkoxide Chemistry Revisited: Synthesis, Structures,
and Characterization of Several Aluminum Alkoxide and Siloxide Complexes. J. Am. Chem. Soc..

[44] Van
de Kerckhove K., Barr M. K. S., Santinacci L., Vereecken P. M., Dendooven J., Detavernier C. (2018). The Transformation
Behaviour of “Alucones”, Deposited by Molecular Layer
Deposition, in Nanoporous Al2O3 Layers. Dalton
Trans..

[45] Liang X., Evanko B. W., Izar A., King D. M., Jiang Y.-B., Weimer A. W. (2013). Ultrathin Highly
Porous Alumina Films Prepared by Alucone
ABC Molecular Layer Deposition (MLD). Microporous
Mesoporous Mater..

