# Supplementary material for: Elucidating the Kinetics and Thermodynamics of Organic Vapor Phase Infiltration and Molecular Layer Deposition for Dissolution Resilient Polymers
Source: Chem Mater. 2025 Oct 3;37(22):9105–15. doi: 10.1021/acs.chemmater.5c01522 (PMC12659019; doi:10.1021/acs.chemmater.5c01522)
Supplement: Supplementary file 1 [file cm5c01522_si_001.pdf]

Supplemental Information

Elucidating the Kinetics and Thermodynamics of Organic Vapor Phase Infiltration and Molecular Layer Deposition for Dissolution Resilient Polymers

Brian C. Welch [a,b], Bratin Sengupta [b,c], Ruoke Cai [d], Vepa Rozyyev [b], Eitan I. Feldman [a,e], Anil Mane [b], Alon Grinberg Dana [a], Jeffrey W. Elam [b], and Tamar Segal-Peretz [a*]*

[a] Department of Chemical Engineering Technion – Israel Institute of Technology, Haifa 3200003, Israel

[b] Applied Materials Division, Argonne National Laboratory, Lemont, Illinois 60439, USA

[c] Northwestern Center for Water Research, Northwestern University, Evanston, Illinois 60201, USA

[d] The Interdisciplinary Program in Polymer Engineering, Technion – Israel Institute of Technology, Haifa, 3200003, Israel

[e] Department of Chemical and Biomolecular Engineering, Rice University, Houston, Texas 77005, USA

*Email bw@campus.technion.ac.il, tamarps@technion.ac.il

I. Tabulated Experimental Information

Table S1. Spin Coater Conditions

polymer	solution wt. %	solvent	temperature - °C	spin rate - rpm	duration - s	film thickness - nm
Mowiol 4-88	2.0	water	90	3000	60	~40
Mowiol 8-88	2.0	water	90	6000	60	~67
PS	1.1	toluene	25	1500	60	~58
PMMA	2.0	toluene	25	3000	60	~54

Table S2. Reaction Conditions* for *In-Situ* Ellipsometry Measurements and Ex-Situ FTIR Samples

substrate	precursor 1	temp. - °C	dose time - s	purge time - s	precursor 2	temp. - °C	dose time - s	purge time - s
spin-coated polymer	IC	130	5	325	MPD	50	5	325
spin-coated polymer	PDIC	85	5	325	MPD	50	5	325
spin-coated polymer	TA	85	5	325	MPD	50	5	325
uncoated Si	IC	130	5	325	MPD	50	5	325
uncoated Si	PDIC	85	5	325	MPD	50	5	325
uncoated Si	TA	85	5	120	MPD	50	5	120

*Reactions were performed at 130°C with a N2 flow of 50 sccm.

Table S3. Thickness of Polymer Substrates* Prior to First Precursor Exposure During *In-Situ* Ellipsometry

substrate	chemistry	original thickness - nm	net growth after 10 cycles - nm	
PVA	IC-MPD	74	4.4	(+5.9%)
PVA	PDIC-MPD	97	0.8	(+0.9%)
PVA	TA-MPD	83	0.2	(+0.2%)
PMMA	IC-MPD	53	-0.04	(-0.07%)
PMMA	PDIC-MPD	52	-0.09	(-0.2%)
PMMA	TA-MPD	53	0.01	(+0.02%)
PS	IC-MPD	65	0.2	(+0.4%)
PS	PDIC-MPD	66	0.09	(+0.1%)
PS	TA-MPD	65	0.05	(+0.09%)

*The samples correspond to the measurements shown in Figure 3a.

Table S4. Ellipsometry Model Parameters

film	model	parameters
PVA*	Cauchy	A = 1.5, B = 0.015 μm^2
PS	Sellmeier	B_1 = 1.4435, C_1 = 0.020216 μm^2
PMMA	Sellmeier	B_1 = 0.99654, C_1 = 0.00787 μm^2, B_2 = 0.18964, C_2 = 0.02191 μm^2, B_3 = 0.00411, C_3 = 3.8573 μm^2
MLD films	Cauchy	A = 1.5, B = 0.015 μm^2

*For dissolutions tests (Figure 7) A and B parameters were fit to PVA samples to extract refractive indices

Table S5. “Short Exposure” Reaction Conditions* for In-Situ QCM Measurements on PVA-Coated Si

precursor 1	temp - °C	dose time - s	purge time - s	precursor 2	temp - °C	dose time - s	purge time - s	N_2 flow - sccm	PVA thickness - nm
IC	130	0.5	330	MPD	130	0.5	330	20	28
PDIC	130	1	330	MPD	130	1	330	20	25
TMA	25	0.3	330	H_2O	25	0.3	330	200	31

*Reactions were performed at 130°C. PVA thickness was measured after MLD processing.

Table S6. “Long Exposure” Reaction Conditions* for In-Situ QCM Measurement on PVA-Coated Si

precursor 1	temp - °C	exposure time - s	partial pressure - Torr	purge time - s	PVA thickness - nm
IC	130	3600	1.3	3600	39
PDIC	130	3600	4.6	3600	42
TMA	25	3600	1.7	3600	36

*All reactions were performed at 130°C in static exposure conditions (no background N_2 flow or pumping). N_2 flow of 100 sccm was used throughout the purges with active pumping.

Table S7. Film Thickness Data for Dissolution Tests

sample		unmodified PVA	Heated PVA	IC-MPD, 10 cycles	PDIC-MPD, 10 cycles	TMA-H_2O, 10 cycles
original state	thickness -nm	53.4	31.3	22.7	38.5	37.0
	refractive index	1.49	1.52	1.53	1.53	1.50
	mean square error	8.2	4.5	3.2	5.6	3.8
after ultrapure water bath	thickness -nm	0	4.9	23.9	13.6	22.0
	refractive index	-	1.63	1.54	1.62	1.51
	mean square error	3.6	1.6	1.3	3.3	1.5
after pH 9 KOH solution bath	thickness -nm	0	5.2	24.8	8.0	18.8
	refractive index	-	1.57	1.54	1.71	1.92
	mean square error	9.2	1.5	2.9	3.3	30
after pH 13 KOH solution bath	thickness -nm	-	0.5	25.7	3.2	13.4
	refractive index	-	1.90	1.51	2.19	1.77
	mean square error	-	1.4	3.1	4.8	3.8

II. Group Additivity Calculation Basis

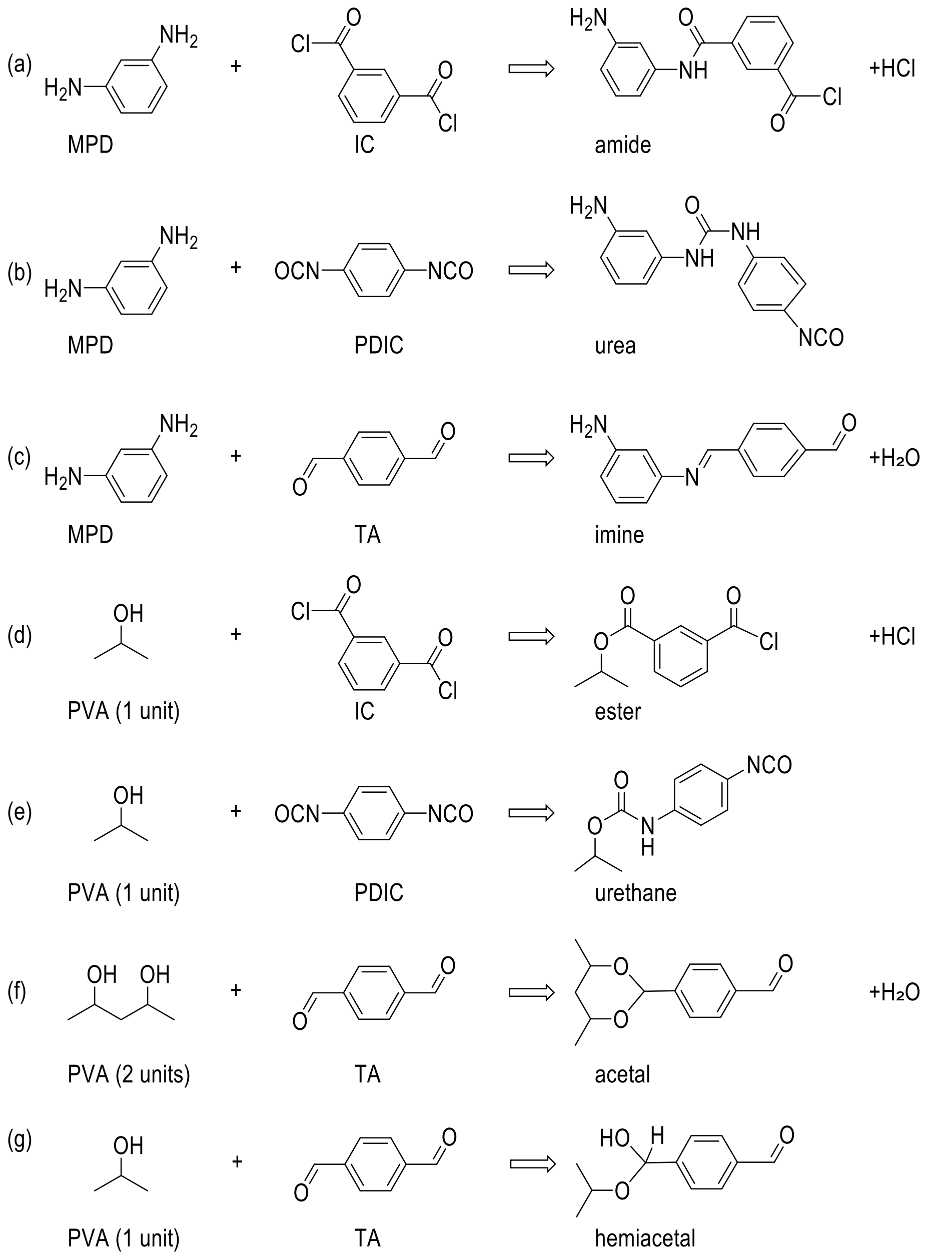

Figure S1. Stoichiometrically balanced reaction schemes which served as the basis for the group additivity calculations.

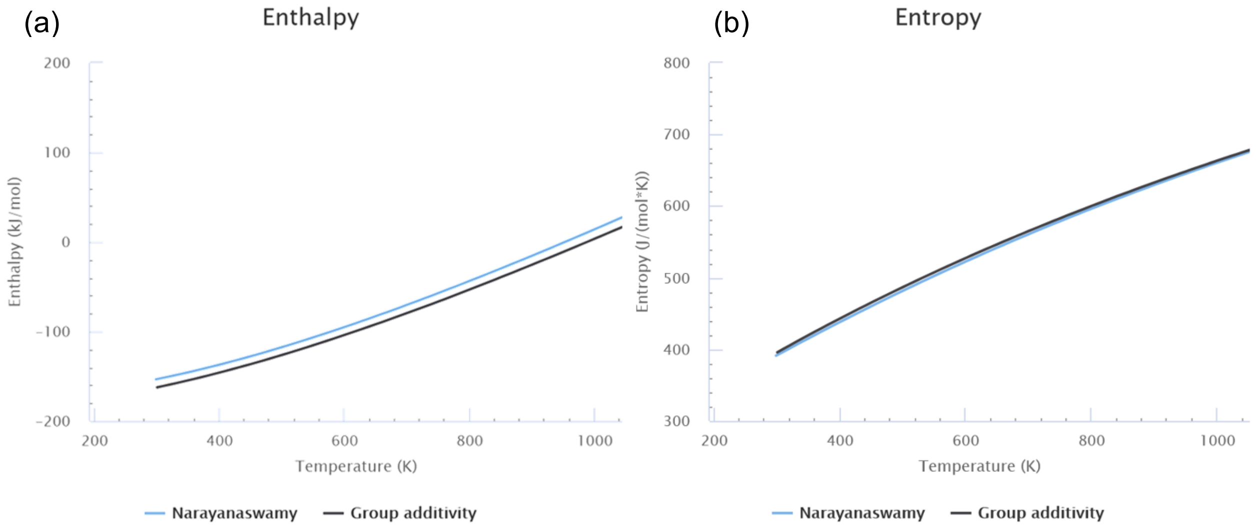

Figure S2. Benchmarking of thermodynamic properties for TA. “Narayanaswamy” is taken from Ref. 1. “Group additivity” is the present work.

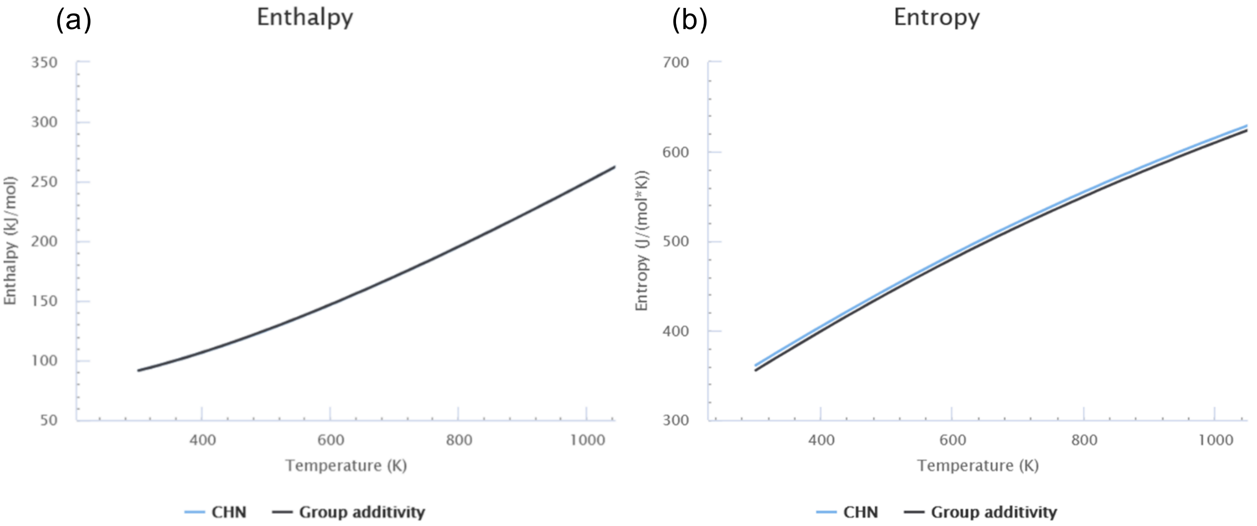

Figure S3. Benchmarking of thermodynamic properties for MPD. “CHN” is a compiled thermodynamic properties source of C/H/N molecules taken from Ref. 2. “Group additivity” is the present work.

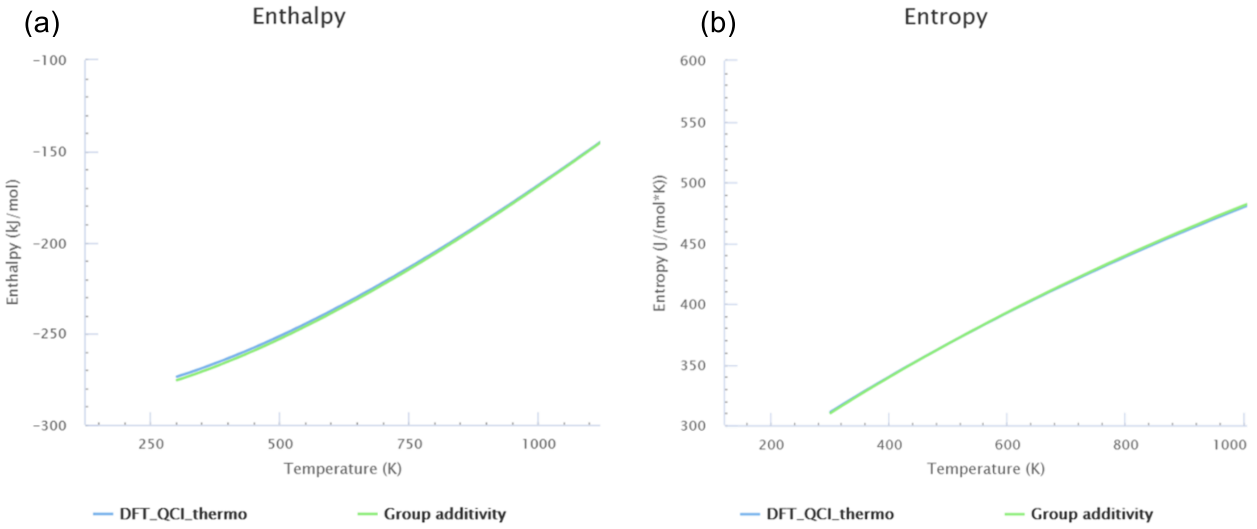

Figure S4. Benchmarking of thermodynamic properties for PVA. “DFT_QCI_thermo” is a compiled thermodynamic library from the Reaction Mechanism Generator database, Ref. 3. “Group additivity” is the present work.

III. XPS Depth Profiles

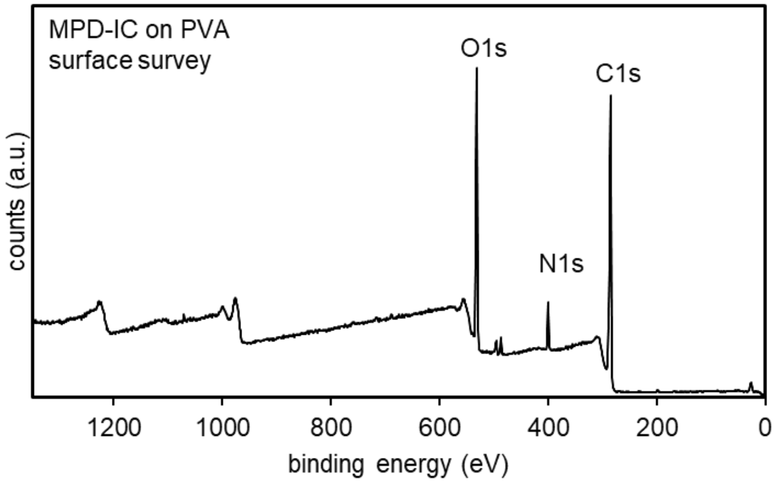

Figure S5. XPS surface survey spectrum of a PVA sample modified with 10 cycles of IC-MPD (5 s doses / 325 s purges).

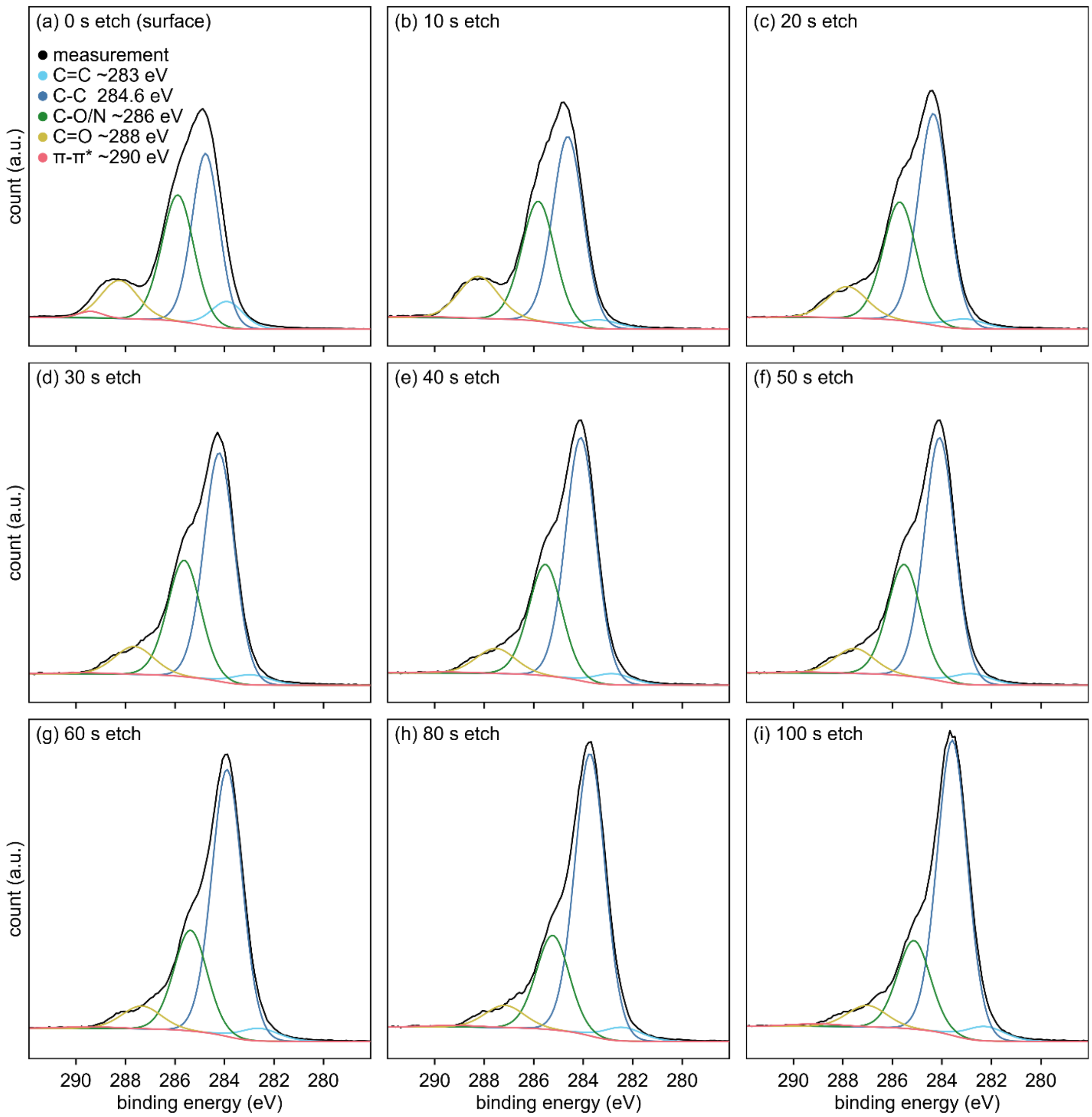

Figure S6. Deconvolution curves of the C 1s peak for various etch depths of a PVA sample treated with 10 cycles of IC-MPD (5 s doses / 325 s purges). These data correspond to the sample and measurements of Figure S7a.

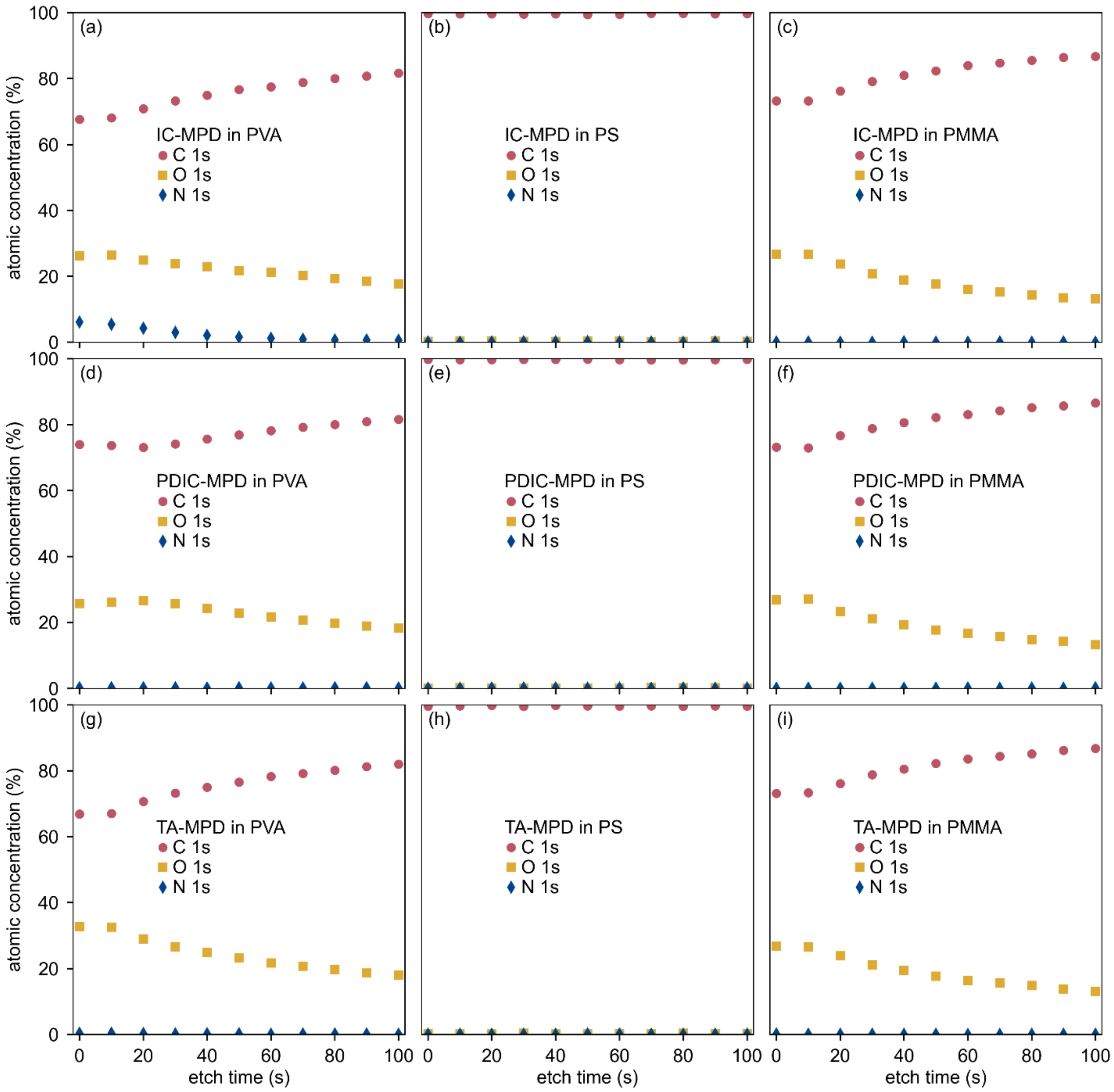

Figure S7. Depth profiles for spin-coated polymer films modified with MLD/VPI. (a)-(c) IC-MPD 5-325-5-325 s recipe at 130°C. (d)-(f) PDIC-MPD 5-325-5-325 s recipe at 130°C. (g)-(i) TA-MPD 5-325-5-325 s recipe at 130°C. A 100s etch time corresponds with an approximate depth of 5 nm.

IV. XPS Depth Calibration

The XPS depth profiling measurements record the XPS signals versus the etch time. To convert etch time into etch depth, we need to know the etch rate. To determine the etch rate of the polymer films, we performed XPS depth profiling measurements on polymer films of known thickness and compared these measurements to simulated depth profiles calculated using

different etch rates. The red trace in Figure S8a shows the normalized Si 2p XPS signal measured during XPS depth profiling of a 28 nm PVA sample with 10 cycles of IC-MPD on a silicon substrate using a high etch current. The Si XPS signal is attenuated during early etch time due to attenuation of the photoelectrons by the modified PVA overlayer. The thickness of this layer can be estimated using the relation:

$$I = I_0 e^{\frac{-d}{\lambda}} \tag{1}$$

Where I is the Si XPS signal at depth d, I_0 is the Si XPS intensity at the surface, and λ is the inelastic mean free path (IMFP) for the XPS photoelectrons through the overlayer. Taking an average IMFP through polymers for 1000 eV electrons of 6.0 (± 1.0) nm,[4] and assuming an $E^{1/2}$ energy dependence for the IMFP,[5] λ = 1.89 (± 0.33) nm at the Si 1p energy of 100 eV. The blue trace in Figure S2 shows simulated Si XPS signals calculated using Eqn. 1 assuming an etch rate of 0.65 nm/s. The XPS data shows a more gradual slope compared to the model, and we believe this results from surface roughness effects.[6] To determine the true etch rate, we calculated model Si XPS signals using a range of etch rate values and calculated the mean squared error (MSE) between the model and the measurements (Figure S8b). We found that the MSE was minimized at an etch rate of 0.65 ± 0.07 nm/s.

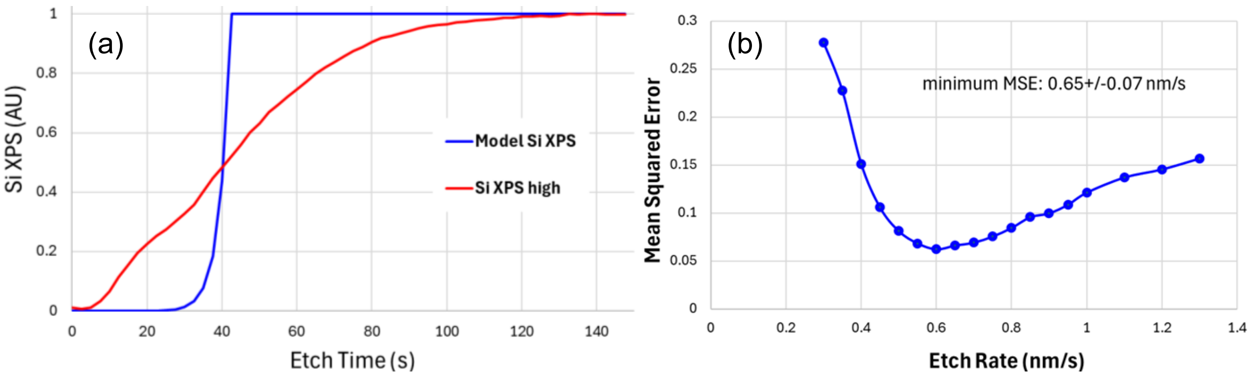

Figure S8. (a) Normalized XPS depth profile for Si 2p peak from 28 nm PVA sample with 10 cycles of IC-MPD on Si (blue) along with modeled Si XPS data assuming etch rate of 0.65 nm/s (red). (b) Mean squared error between model Si XPS signals and measured Si XPS.

The measurements in Figure S8b were performed using a high etch current in order to completely remove the polymer overlayer. However, the XPS depth profiling experiments in Figure 3b were performed using a low etch current to achieve a higher depth resolution, but this approach did not completely remove the polymer layer. Consequently, the etch rate cannot be determined from these measurements using the method described above. However, the relative etch rate using the low etch current can be evaluated by comparing the etch times required to achieve the same Si 2p XPS signal for the high and low etch currents. Figure S9 shows the XPS depth profiling measurements of Si 2p peak from a 25 nm PVA sample with 10 cycles of PDIC-MPD on Si measured using high (red) and low (blue) etch currents. The XPS data are both normalized to the saturation Si signal recorded using the high etch current. Figure S9 shows that the Si XPS signal is 0.33 (a.u.) after 300s etch time using the low etch current. This same signal level is achieved using 24 s with the high etch current. Consequently, the etch rate using the low etch current is: 0.65 ± 0.07 nm/s × (24/300) = 0.052 ± 0.006 nm/s.

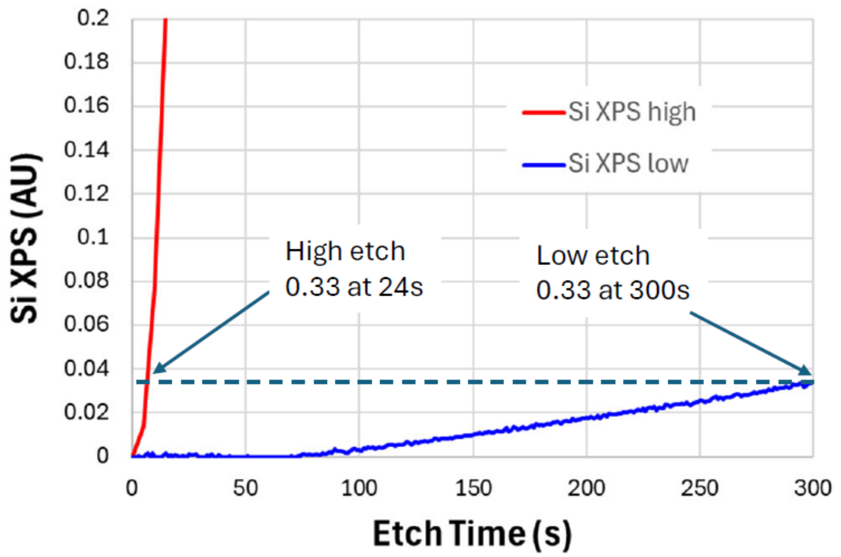

Figure S9. XPS depth profiling measurements of the Si 2p peak of 25 nm PVA sample with 10 cycles of PDIC-MPD on Si measured using high (red) and low (blue) etch currents. The XPS data are both normalized to the saturation Si signal using the high etch current.

V. Reaction-Diffusion Model

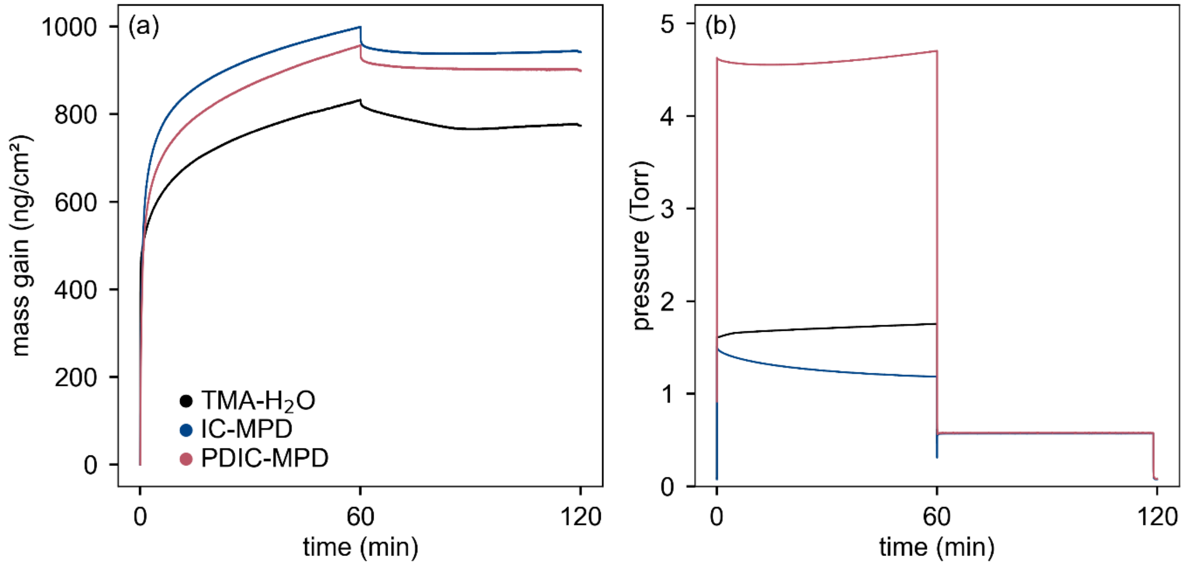

Figure S10. (a) Mass gain of PVA films measured by QCM and (b) pressure data for the long exposure experiments (1 h doses / 1 h purges) described in

Table S6. Precursor exposure occurred in the first 60, and purging occurred in the last 60 min. The normalized data in Figure 5a–c are derived from (a).

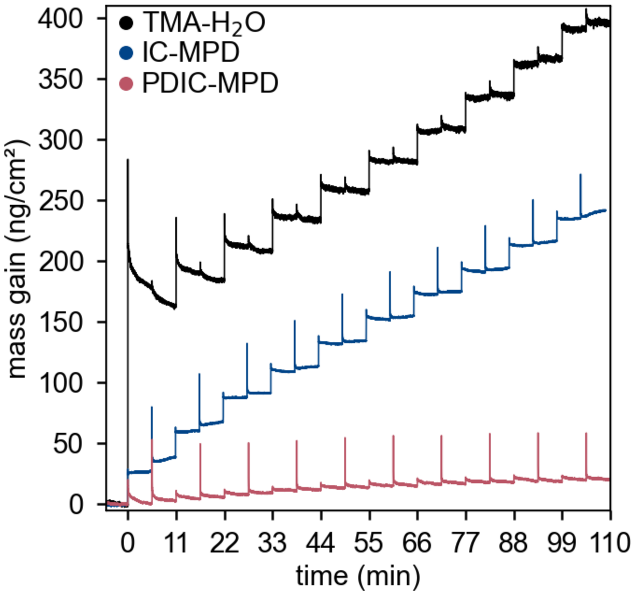

Figure S11. Mass gain of PVA films measured by QCM for the short exposure experiments (≤ 1 s doses / 330 s purges) described in *For dissolutions tests (Figure 7) A and B parameters were fit to PVA samples to extract refractive indices Table S5. The normalized data in Figure 5d–f are derived from the first 330 s of these data.

Fitting D, Diffusion Coefficient

To estimate the initial diffusion coefficients, D_0, we fit a Fickian diffusion model to the mass gain data obtained during the initial precursor exposure, measured with QCM (Figure 5a-c). The fit was applied to only the initial few milliseconds of data, based on the assumption that precursor diffusion was unhindered by reaction during the initial moments. This assumption holds most accurately when the reaction rate, k, and the diffusion hindering parameter, ℸ, are low. Previous studies have demonstrated its validity for TMA-PMMA systems.[7,8] The diffusion equation and associated initial and boundary conditions were

$$\partial C/\partial t = D_0 \cdot \partial^2 C/\partial x^2 \tag{2}$$

$$C = 0 \text{ for } -l < x < l \text{ at } t = 0 \tag{3}$$

$$C = C_S \text{ for } t > 0 \text{ at } x = \pm l \tag{4}$$

$$\partial C/\partial x = 0 \text{ for } t > 0 \text{ at } x = 0 \tag{5}$$

where D (cm^2/s) is diffusivity of the precursor in the polymer, and C is precursor concentration at time t (s) at a depth of x (cm) into polymer of thickness $2l$ (cm). This models diffusion from both sides, which is mathematically identical to our experiment of a one-sided system of thickness l. The analytical solution[9,10] for this one-dimensional system is

$$\frac{m_t}{m_\infty} = 1 - \sum_{n=0}^{\infty} \frac{8}{(2n+1)^2\pi^2} \cdot \exp\left(\frac{-D(2n+1)^2\pi^2 t}{4l^2}\right) \tag{6}$$

where m_t/m_∞ is the instantaneous mass uptake normalized to the saturated mass uptake. We approximated m_t/m_∞ by calculating Eqn. (6) to the fourth term ($n = 4$).

Reaction Diffusion Model

Ren et al. developed a transport model (the "Ren-McGuinness model") to account for Fickian diffusion along with species depletion through reaction and a diminishing diffusion

coefficient due to transport resistance from incorporated material.[9] The model relies on the following equations, calculated numerically based on source code published on Github[11]

$$\frac{\partial C_{free}}{\partial t} = D\frac{\partial^2 C_{free}}{\partial x^2} - k\, C_{free}\, C_{polymer} \quad (7)$$

$$D = D_0\, exp\,(-K'\, C_{product}) \quad (8)$$

$$\frac{\partial C_{prod}}{\partial t} = k\, C_{free}\, C_{polymer} \quad (9)$$

$$\frac{\partial C_{polymer}}{\partial t} = -k\, C_{free}\, C_{polymer} \quad (10)$$

where:

- C_{free}: concentration of absorbed, unreacted precursor
- $C_{polymer}$: concentration of accessible substrate functional groups that have not reacted
- $C_{product}$: concentration of functional groups that reacted with precursor
- D_0: diffusion coefficient before VPI reactions
- K': diffusion hindering factor describing reduced diffusivity after reaction

The initial and boundary conditions are

$C_{free} = 0$ for $-l < x < l$ at $t = 0$ (11)

$C_{product} = 0$ for $-l < x < l$ at $t = 0$ (12)

$C_{polymer} = C^0_{polymer}$ for $-l < x < l$ at $t = 0$ (13)

$C_{free} = C_S$ for $0 < t < 3600\, s$ at $x = \pm l$ (14)

$C_{free} = 0$ for $3600 < t < 7200\, s$ at $x = \pm l$ (15)

$\partial C_{free}/\partial x = 0$ for $t > 0$ at $x = 0$ (16)

where $C^0_{polymer}$ is the initial concentration of accessible substrate functional groups. This system models diffusion from both sides of a film with thickness $2l$, which is mathematically identical to our experiment of a one-sided system of thickness l.

Calculation of $\boldsymbol{C_s}$ and $\boldsymbol{C^0_{polymer}}$

At the end of a 1 h exposure to a precursor (Figure 5a-c), it was assumed that all available reaction sites of the polymer film had reacted. Therefore, the net mass gain after exposure (m_A) (t = 3600 s) was equal to the sum of reacted precursor (molar mass, M_{net}) and unreacted, absorbed precursor (molar mass, M) equilibrated to the surface concentration of C_S, calculated as

$$C_S = (m_B - m_A)/(l\,M) \tag{17}$$

After the 1 h purge, all unreacted precursor was assumed removed. Therefore, the net mass gain at the end of the purge (m_B) (t = 7200 s) was related to $C^0_{polymer}$ as

$$C^0_{polymer} = m_B/(l\,M_{net}) \tag{18}$$

It was assumed that each precursor reacted once, and the mass gain, M_{net}, was equal to the precursor's molar mass minus the molar mass of a single reaction byproduct (M_{net} for TMA = 56.04 g/mol; IC = 166.56 g/mol; PDIC = 160.13 g/mol). The PDIC-hydroxyl reaction produces no reaction byproducts, so $M_{net} = M$ for PDIC. For IC and TMA, M_{net} is effectively lower if precursor undergoes multiple reactions within the polymer. However, the fitting of Da and ℸ is not sensitive to the value of M_{net}. Byproducts from TMA-acetate reactions and multiple reactions were disregarded.

Ren et al. originally used $M_{net} = M =$ 72.09 g/mol for TMA in PMMA.[9] To correct their results for mass loss from the ethane byproduct, we recalculated M_{net} as the molar mass of TMA minus ethane ($M_{net} =$ 42.02 g/mol).[12] After fitting their data, $C^0_{polymer}$ was adjusted by a factor of

(72.09/42.02), while K', k, and Kaf were adjusted by a factor of (42.02/72.09), as summarized in Table S7. All other parameters remained unchanged to maintain the equivalent fitting of their data.

Table S8. Ren-McGuinness Model Results Before and After Correcting for Mass Loss of Reaction Byproducts

	M_{net}	K'	k	D	$C^0_{polymer}$	Cs	l	Da	כ	ר
	g/mol	cm³/mol	cm³/mol·s	cm²/s	mol/cm³	mol/cm³	nm			
uncorrected results[9]	72.09	1150	1	1.65×10^{-10}	5.7×10^{-3}	4.4×10^{-3}	480	0.08	0.78	6.5
corrected results	42.02	670	0.6	1.65×10^{-10}	9.7×10^{-3}	4.4×10^{-3}	480	0.08	0.45	6.5

Fitting $\boldsymbol{k}$ (Reaction Rate) and $\boldsymbol{K'}$ (Hindering Factor)

The hindering factor, K', was determined by fitting the model to QCM measurements of mass loss due to desorption during the 1 h purge step (t = 3600 to 7200 s, Figure 5a-c). In the original study, Ren et al. fit the reaction rate, k, "subjectively" based on the authors' judgement.[9] However, the k can be estimated more objectively by fitting the model to second experiment involving short precursor exposure (<1 s) and moderate purge times (~10 min), as shown in Figure 5d-f.

With minimal exposure, the hindering factor (K') has reduced influence, and the retained mass depends primarily on k. A high k value results in greater mass retention, as observed for IC and TMA in PVA (Figure 5d,e). Conversely, a low k value leads to noticeable mass loss through desorption, as seen for PDIC (Figure 5f). By iteratively fitting k and K' to both long and short exposure data, we obtained estimates for both parameters.

VI. Estimation of Molar Increase from Mass Increase

The QCM measurements of Figure S11 provided mass gains for the electrophiles and MPD. To calculate the molar gains for the electrophiles, we must assess whether the precursor reacted once, yielding a single byproduct molecule, or twice, yielding two, according to

$$n_E = x\frac{m_E}{M_E - M_{BP}} + (1-x)\frac{m_E}{M_E - 2M_{BP}} \quad (19)$$

Here, m_E is the total mass increase from all electrophile dose steps, n_E is the corresponding molar increase, x is the fraction of single reactions, M_E is the molar mass of the electrophile, and M_{BP} is the molar mass of the byproduct. Note that for the PDIC-MPD, which has no byproducts,

$$n_E = \frac{m_E}{M_E} \quad (20)$$

To estimate the fraction of single reactions (x), the following assumptions were made:

1. MPD only had single reactions because they could not react with the substrate, thus the total molar increase from MPD steps (n_{MPD}) could be related to its total mass increase (m_{MPD}) according to

$$n_{MPD} = \frac{m_{MPD}}{M_{MPD} - M_{BP}} \quad (21)$$

2. Each electrophile that had a single reaction subsequently reacted with MPD.

$$n_{MPD} = x\frac{m_E}{M_E - M_{BP}} \quad (22)$$

$$x = \frac{n_{MPD}}{m_E}(M_E - M_{BP}) \quad (23)$$

References

(1) Narayanaswamy, K.; Blanquart, G.; Pitsch, H. A Consistent Chemical Mechanism for Oxidation of Substituted Aromatic Species. *Combustion and Flame* **2010**, *157* (10), 1879–1898. https://doi.org/10.1016/j.combustflame.2010.07.009.

(2) Yaws, C. L. *Yaws' Critical Property Data for Chemical Engineers and Chemists*; Knovel: Norwich, N.Y, 2012.

(3) Johnson, M. S.; Dong, X.; Grinberg Dana, A.; Chung, Y.; Farina, D. Jr.; Gillis, R. J.; Liu, M.; Yee, N. W.; Blondal, K.; Mazeau, E.; Grambow, C. A.; Payne, A. M.; Spiekermann, K. A.; Pang, H.-W.; Goldsmith, C. F.; West, R. H.; Green, W. H. RMG Database for Chemical

Property Prediction. *J. Chem. Inf. Model.* **2022**, *62* (20), 4906–4915. https://doi.org/10.1021/acs.jcim.2c00965.

(4) Cadman, P.; Gossedge, G.; Scott, J. D. The Determination of the Photoelectron Escape Depths in Polymers and Other Materials. *Journal of Electron Spectroscopy and Related Phenomena* **1978**, *13* (1), 1–6. https://doi.org/10.1016/0368-2048(78)85001-4.

(5) Seah, M. P.; Dench, W. A. Quantitative Electron Spectroscopy of Surfaces: A Standard Data Base for Electron Inelastic Mean Free Paths in Solids. *Surface and Interface Analysis* **1979**, *1* (1), 2–11. https://doi.org/10.1002/sia.740010103.

(6) Shard, A. G.; Baker, M. A. Practical Guides for X-Ray Photoelectron Spectroscopy: Use of Argon Ion Beams for Sputter Depth Profiling and Cleaning. *Journal of Vacuum Science & Technology A* **2024**, *42* (5), 050801. https://doi.org/10.1116/6.0003681.

(7) Caligiore, F. E.; Nazzari, D.; Cianci, E.; Sparnacci, K.; Laus, M.; Perego, M.; Seguini, G. Effect of the Density of Reactive Sites in P(S-r-MMA) Film during Al2O3 Growth by Sequential Infiltration Synthesis. *Advanced Materials Interfaces* **2019**, *6* (12), 1900503. https://doi.org/10.1002/admi.201900503.

(8) Perego, M.; Motta, A.; Ronnby, K.; Yap, F. T. J.; Seguini, G.; Wiemer, C.; Nolan, M. On the Differences in Trimethylaluminum Infiltration into PMMA and PLA Polymers for Sequential Infiltration Synthesis: Insights from Experiment and First Principles Simulations. ChemRxiv November 4, 2024. https://doi.org/10.26434/chemrxiv-2024-zdk5k.

(9) Ren, Y.; McGuinness, E. K.; Huang, C.; Joseph, V. R.; Lively, R. P.; Losego, M. D. Reaction–Diffusion Transport Model to Predict Precursor Uptake and Spatial Distribution in Vapor-Phase Infiltration Processes. *Chem. Mater.* **2021**, *33* (13), 5210–5222. https://doi.org/10.1021/acs.chemmater.1c01283.

(10) Crank, J. *The Mathematics of Diffusion*, 2nd ed.; Clarendon Press: Oxford, 1975.

(11) Huang, C.; Yi, R.; Joseph, R. V.; Lively, R. P.; Losego, M. D. VPI Reaction-Diffusion Model Simulation Code, 2023. https://github.com/Losego-Lab/Reaction-Diffusion_Transport_Model (accessed 2024-01-19).

(12) Waldman, R. Z.; Mandia, D. J.; Yanguas-Gil, A.; Martinson, A. B. F.; Elam, J. W.; Darling, S. B. The Chemical Physics of Sequential Infiltration Synthesis—A Thermodynamic and Kinetic Perspective. *The Journal of Chemical Physics* **2019**, *151* (19), 190901. https://doi.org/10.1063/1.5128108.
